# Formulation Optimization and Quality Evaluation of Walnut Protein Sausage Based on Fuzzy Mathematics Sensory Evaluation Combined With Random Centroid Optimization

**DOI:** 10.1002/fsn3.70747

**Published:** 2025-08-17

**Authors:** Ying Wang, Chengzhuang Lv, Ping Zhang, Fengzhong Wang, Ting Zhang, Yan Ma, Mingqiang Xu

**Affiliations:** ^1^ Institute of Agro‐Products Processing Xinjiang Academy of Agricultural Sciences Urumqi China; ^2^ Xinjiang Research Center for Deep Processing Engineering of Major Agricultural and Sideline Products Urumqi China; ^3^ Institute of Food Science and Technology Chinese Academy of Agricultural Sciences Beijing China

**Keywords:** fuzzy mathematics sensory evaluation, protein sausage, quality, random centroid mapping, walnut meal

## Abstract

To explore new plant protein resources and develop novel walnut‐based protein products, sensory scores were adopted as an evaluation index, and seven optimization factors, which included salt, sugar, modified starch, egg white powder, walnut oil, lab‐prepared spices, and walnut tissue protein, were selected. A fuzzy mathematics sensory evaluation model integrated with random centroid optimization was developed to optimize the processing parameters of walnut protein sausage. A comparative analysis was conducted, with soy protein sausages, wheat protein sausages, and commercially available vegetarian sausages, based on the basic physicochemical properties, texture characteristics, nutritional functions, and biomimetic sensory qualities of walnut protein sausages. The results demonstrated that the optimal formulation for walnut protein sausage comprised 1.8% salt, 3.4% sugar, 7% modified starch, 5.1% egg white powder, 1.8% walnut oil, 0.8% lab‐prepared spices, and 66.1% walnut protein. Walnut protein sausages prepared with this formula had the highest sensory score (92.01 points). The prepared walnut protein sausages had a distinct walnut aroma, smooth surface, uniform and firm texture, and compact structure. The sausages were high in dietary fiber (7.40 g/100 g), low in crude fat (6.59 g/100 g), and demonstrated good water‐holding capacity (7.78%) and oil‐holding capacity (19.92%). Pearson correlation analysis revealed significant relationships between the physicochemical, functional, and nutritional properties of various vegetarian sausages, with indicators such as crude protein, crude fat, and moisture content closely associated with flavor. The walnut‐based sausages met the food safety requirements for healthy products. This study facilitates the development of plant‐based vegetarian sausages from walnut protein, which in turn increases the added value of walnut by‐products.

## Graphical abstract

To explore new sources of plant protein and develop new walnut and vegetarian meat products, a sausage with a walnut aroma, smooth surface, uniform body, and compact structure was developed using a high‐moisture extruder.

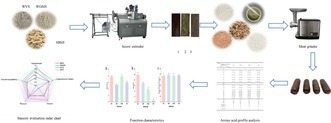

## 1 Introduction

With the increase in demand for more diverse and healthy diets, alongside concerns about the quality, safety, and sustainability of meat production, plant‐based protein products have emerged as optimal meat substitutes. However, only a few plant protein sources meet the requirements for optimal meat substitutes. Thus, the development of multi‐source plant protein products with promising market applications represents a novel approach in food innovation (Sha and Xiong 2020). Walnut, which has the largest cultivation area and production in China, is one of the major woody oil crops actively promoted by the government. Walnut cultivation, owing to its several unique advantages, also serves as a key industry for poverty alleviation among farmers in Xinjiang, China. By 2022, walnut was the second most cultivated crop in China, with plantations in Xinjiang spanning > 6.5 million acres (~433,333 ha) of land and production exceeding 1.2 million tons. Walnut processing in Xinjiang currently presents challenges such as short industry chains, inefficient utilization of by‐products, and low added value. Specifically, defatted walnut meal, a by‐product of oil extraction, is either sold as low‐cost animal feed or discarded (Kong Ling‐Ming et al. 2013). A previous study found that the protein content of walnut meal was > 40% and the main protein component was gluten, which accounted for 79.82% of total walnut protein (Xie et al. 2023). This composition was significantly different from that of soy protein and pea protein fractions, which were dominated by 7 S and 11 S globulins and had a protein content of 35%–40% (Wu et al. 2024). For amino acid composition, walnut meal contains 20 amino acids, which include 8 essential amino acids. The fatty acid composition of walnut meal has the highest linoleic acid content (61.71%), and the proportion of α‐linolenic acid (ALA) is 10%–15%. This ALA content is much higher than that of soybeans and peas, which are dominated by omega‐6 fatty acids. Furthermore, the total dietary fiber content of walnut meal is 15%–20%, which is significantly higher than that of soybean (10%–15%) and pea (8%–12%). In addition, walnut meal contains ellagic acid, gallic acid, and other polyphenols. Its antioxidant activity (ORAC value) is two to three times that of soybean meal. Thus, walnut meal can be used as a natural antioxidant in food and in other industries (Ma et al. 2024). These nutritional properties, especially the high protein content, indicate that the nutritional value of walnut meal is significantly better than that of soybean/pea resources. Thus, walnut meal can be used as a novel plant protein resource.

Protein sausages are vegetarian products predominantly made from plant protein via modern food processing techniques (such as extrusion and steam cooking) to simulate the flavor and texture of meat. Current research on protein sausages focuses mainly on formulation optimization and quality evaluation. Typical plant protein products include pea and chickpea protein sausages, jackfruit sausages, and low‐fat oatmeal sausages. No reports exist in the literature of sausages made from walnut meal as a protein base. Noguerol et al. (2022) pea protein and chickpea protein were used as the main ingredients for protein sausages and optimized the effect of plantain fiber on composition, physicochemical properties, and mechanical characteristics via orthogonal methods. Keerthana Priya et al. (2022) used green jackfruit and banana flower as the main ingredients for protein sausages, optimized the processing technology and formulation of jackfruit sausages via orthogonal experiments, and evaluated the texture, physicochemical properties, and sensory characteristics. Kerr et al. (2005) prepared low‐fat Italian sausages using hydrated oatmeal as the main ingredient, analyzed the texture profile and color, and conducted consumer sensory evaluations via response surface methodology. However, the subjective nature of traditional sensory evaluations makes the quantification of results difficult. Moreover, orthogonal and response surface optimization methods are labor‐intensive, time‐consuming, and expensive. In contrast, random centroid optimization (RCO) directly optimizes multiple factors without the need for single‐factor optimization experiments. The RCO method facilitates global optimization, which reduces the number of trials and improves efficiency. The application of fuzzy mathematics in sensory evaluation eliminates subjective assessment errors, which in turn enables an objective and accurate reflection of the importance of various factors. This process enhances the scientific, rational, and objective nature of sensory evaluation results.

This study optimizes the processing technology and formulation of walnut protein sausages via a combined fuzzy mathematics sensory evaluation and RCO method. The study analyses quality indicators such as the texture, physicochemical properties, and functional characteristics of the product to develop a “green, nutritious, safe, and healthy” walnut protein sausage. This study provides a novel approach for the efficient utilization of walnut by‐products. This work holds significant practical value as it provides insights into the efficient utilization of walnut meal, improves the added value of by‐products, extends the walnut processing industry chain, alleviates resource pressure, and facilitates new developments within the walnut processing industry.

## 2 Materials and Methods

### 2.1 Materials and Reagents

Walnut protein sausages, soy protein sausages, and wheat protein sausages were prepared in the lab. Commercially available salt, granulated sugar, modified starch, egg white powder, walnut oil, and protein sausage casings were purchased at Beiyuanchun Market in Urumqi, Xinjiang. Analytical grade reagents: sulfuric acid was obtained from Sichuan Xilong Scientific Co. Ltd., while copper sulfate, potassium sulfate, petroleum ether, hydrochloric acid, phenol, nitrogen gas, sodium citrate, and sodium hydroxide were obtained from Tianjin Zhiyuan Chemical Reagent Co. Ltd.

### 2.2 Instruments and Equipment

The equipment used in this study were as follows: Electronic balance, TP‐A2000 model (Huazhi/Fujian Electronic Technology Co. Ltd.); automatic Kjeldahl nitrogen analyzer, K9840 model (Haineng Future Technology Group Co. Ltd.); graphite digestion instrument, SH220F model (Haineng Future Technology Group Co. Ltd.); muffle furnace, MFLXD325‐12 (Shanghai Muffle Furnace Technology Instrument Co. Ltd.); moisture analyzer, MA35M model (Sartorius GmbH, Germany); ultrafine grinder, IKA A11 basic (Xiamen Guangshengda Instrument Equipment Co. Ltd.); centrifuge, TCL‐20bR (Zhejiang Saide Instrument Equipment Co. Ltd.); colorimeter, CR‐400 (Shenzhen Lisun Precision Instrument & Equipment Co. Ltd.); texture analyzer, PILOT (Beijing Yingsheng Hengtai Technology Co. Ltd.); sausage stuffer, THMGF350A (Guangdong Shundetenghui Electrical Appliances Co. Ltd.); twin‐screw extruder, FMHE22 (Hunan Fuma Technology Co. Ltd.); electric constant‐temperature water bath pot, JOANLAB (Beijing Guangming Medical Instrument Co. Ltd.); electronic tongue, SA402B (Insent Co. Ltd. Japan); electronic nose, PEN3 (AIRSENSE Analytics GmbH Germany); amino acid automatic analyzer, Essentia LC‐16AAA (Qingdao Punuo Technology Co. Ltd.); and amino acid automatic analysis balance, Essentia LC‐16AAA (Qingdao Punuo Technology Co. Ltd.).

### 2.3 Experimental Methods

#### 2.3.1 Preparation of High‐Moisture Walnut Tissue Protein

Defatted walnut meal (60% base material), soy protein isolate (25%), wheat gluten (15%), and a quality improver (1%) were thoroughly mixed and processed using a twin‐screw high‐moisture extruder. The extrusion was performed at a screw speed of 240 r/min, a feed rate of 14.5 kg/h, and an outlet extrusion temperature of 120°C to obtain walnut tissue protein.

#### 2.3.2 Preparation of Walnut Protein Sausage

The walnut protein sausage was prepared as follows: The extruded walnut tissue protein was chopped in a grinder for 30 s and then thoroughly mixed with salt, red koji, modified starch, and flavor enhancers. The mixture was stuffed and tied using an automatic sausage stuffer. The preparation method for control samples was the same, with walnut tissue protein replaced by soy tissue protein and wheat tissue protein separately. The stuffed mixture was steamed for 10 min and sterilized at 121°C for an additional 10 min. After cooling the sausages to room temperature, they were packaged and stored frozen at −20°C.

#### 2.3.3 Sensory Evaluation Methods

A sensory evaluation panel comprising 10 graduate students majoring in food science was established to conduct sensory evaluations, according to the T/CIFST 001‐2020 “Plant‐Based Meat Products” guidelines with slight modifications. The evaluation focused on attributes of walnut protein sausage such as appearance, texture, taste, and flavor. Each attribute was evaluated according to the sensory evaluation criteria outlined in Table 1, with a scoring range of 0–20 points. After evaluating each sample, panelists rinsed their mouths with purified water and observed a 4‐min interval before the next.

**TABLE 1:** Sensory evaluation criteria for walnut protein sausage.

Appearance	Texture	Taste	Flavor	Grade
Uniform color, uniformly full and intact casings, well‐sealed, no oozing contents.	Smooth surface, tightly organized, good elasticity, good slicing.	Comfortable mouthfeel, good elasticity, good chewability.	Salty, light walnut flavor, and no off‐flavor.	Excellent (≥ 90)
Uniform color, more uniform and fuller casings, better sealing, no leakage of contents.	Smoother surface, loose texture, good elasticity, good sectioning.	More comfortable mouthfeel, better elasticity, good chewability.	Better saltiness, lighter walnut flavor, no off‐flavor.	Good (75–89)
More uniform in color, more homogeneous and intact casings, well‐sealed, no oozing contents.	Smooth surface, more loosely organized, fair elasticity, fair sectioning.	Average mouthfeel, average elasticity, average chewability.	Moderately salty, very light walnut flavor, no noticeable off‐flavor.	Fair (60–74)
Uneven color, unevenly full and incomplete casings, average sealing, oozing contents.	Surface not smooth, very loose texture, poor elasticity, poor slicing.	Rough mouthfeel, poor elasticity, poor chewability.	Saltier, no walnut flavor, off‐flavors.	Poor (≤ 59)

#### 2.3.4 Establishment of Fuzzy Mathematical Model

##### 2.3.4.1 Establishment of Factor Set, Evaluation Set, and Weight Set

According to the sensory evaluation method outlined by Liu et al. (2022) the evaluation indicators for walnut protein sausage were defined as appearance, texture, taste, and flavor. These indicators were represented by the factor set: U = [U_1_, U_2_, U_3_, U_4_] = [Appearance, Texture, Taste, Flavor]. The evaluation set was defined as: V = [V_1_, V_2_, V_3_, V_4_] = [Excellent, Good, Fair, Poor].

##### 2.3.4.2 Determination of Factor Set Weights

The “0, 4” scoring method was used to determine the weights of each factor. Panelists conducted pairwise comparisons to evaluate the importance of each factor. According to the perceived differences in importance, they assigned scores: “0:4” for a significant difference, “1:3” for a moderate difference, and “2:2” for similar importance. The total score for each factor was then divided by the sum of scores across all factors to establish the weights of each factor. These weights were represented by the weight set.

According to the statistical results of sensory evaluation for walnut protein sausage, which were determined by the evaluators using standard sensory evaluation indicators, the weight coefficients for appearance, texture, taste, and flavor were defined as the weight matrix AU = [AU_1_, AU_2_, AU_3_, AU_4_]. Ten professionals with expertise in food science and relevant fields were selected to evaluate the importance of the four sensory evaluation factors—appearance, texture, taste, and flavor—of the walnut protein sausage. Table 2 presents the resulting weight set for the walnut protein sausage. The evaluation set was defined as {Excellent, Good, Fair, Poor}.

**TABLE 2:** Weight set of evaluation factors for walnut protein sausage.

Factors	Appearance	Texture	Taste	Flavor
Weights X (%)	26%	26%	22%	26%

##### 2.3.4.3 Establishment of Fuzzy Matrix and Fuzzy Transformation

Fuzzy mathematical methods were used to assess the comprehensive sensory evaluation results. The number of evaluators assigning each grade (Excellent, Good, Fair, Poor) to each sensory indicator was tallied according to the scoring criteria of the sensory evaluation. The evaluation results for each group were calculated based on the principles of fuzzy mathematics and matrix transformation, as follows: L = *R* × AU, where R represents the number of votes for each grade across different factors divided by the total number of evaluators.

#### 2.3.5 Random Centroid Optimization

The RCO program includes random cyclic search, centroid search, and mapping composition (Xin et al. 2022; Nishimura and Saeki 2018). Following the input of variables and corresponding ranges into the RCO program, the optimal parameter values obtained from random searches were used for process optimization. The parameters were input into the first round of the random search program, and the results obtained were further optimized using the centroid search program. The compiled and mapped results provided an optimization map for the experimental rounds. A decision was made, based on the degree of dispersion or clustering of results in the mapping optimization chart, whether to conduct subsequent rounds of experiments or not.

In this experiment, sensory scores of walnut protein sausage were used as evaluation indicators. RCO was applied to optimize the design of seven main factors: salt, sugar, denatured starch, egg white powder, walnut oil, lab‐prepared spices, and walnut tissue protein. Table 3 presents the upper and lower limits of the experimental optimization factors.

**TABLE 3:** Upper and lower limits of optimization factors.

Number of cycles	Factors to be optimized	Edible salt (%)	Sugar (%)	Modified starch (%)	Powdered egg white (%)	Walnut oil (%)	Spice blend (%)	Walnut tissue protein (%)
First round	Factor upper limit	5	5	10	8	2	2	90
	Factor lower limit	1	1	5	1	1	0.5	40
Second round	Factor upper limit	2	4	9	7	2	1	90
	Factor lower limit	1	1	5	1	1	0.5	40

### 2.4 Quality Analysis of Walnut Protein Sausage

#### 2.4.1 Basic Physicochemical Index Determination

##### 2.4.1.1 Color

The sausage was cut into sections (15 × 15 × 10 mm), and the color was evaluated using a handheld colorimeter (L*, a*, b* color space) (Szpicer et al. 2022).

##### 2.4.1.2 Moisture

Moisture content was determined using a halogen moisture analyzer (Heinze and Isengard 2001) as follows: First, ~1 g of the sample was accurately weighed and evenly spread on an aluminum dish. Then, the dish was placed in the moisture analyzer and heated at 105°C.

##### 2.4.1.3 Ash

Ash content was determined using AOAC 940.26 (2000) as follows: ~3 g of the sample was placed in a pre‐weighed crucible, heated to a smokeless state in a high‐temperature furnace, and then incinerated in a muffle furnace at 550°C for 5 h. After cooling to ~200°C, the crucible was removed, cooled in a desiccator for 30 min, and then weighed. The formula for ash content was as follows:

(1) $$X=\frac{m_{1}-m_{2}}{m_{3}-m_{2}}\times 100$$

X represents the ash content of the sample (g/100 g); m_1_ represents the mass of the crucible and ash (g); m_2_ represents the mass of the crucible (g); and m_3_ represents the mass of the crucible and sample (g).

##### 2.4.1.4 Crude Fat

Crude fat content was determined via the Soxhlet extraction method as follows: ~3 g of the sample was placed in the extraction tube of the Soxhlet extractor, and the receiver flask, pre‐dried to constant weight, was filled with sufficient petroleum ether (boiling point 55°C). The flask containing petroleum ether was heated in a water bath, which allowed continuous reflux for 6 h (6 cycles per h). The extracted fat was then recovered, and the receiver flask containing the fat was dried in an oven at 100°C ± 5°C for 1 h to remove any residual petroleum ether, after which it was weighed. The formula for fat content was as follows:

(2) $$X=\frac{m_{1}-m_{0}}{m_{2}}\times 100$$

X represents the fat content of the sample (g/100 g); m_1_ represents the mass of the receiving flask and fat at constant weight (g); m_0_ represents the mass of the receiving flask (g); and m_2_ represents the mass of the sample (g).

##### 2.4.1.5 Crude Protein

Crude protein content was determined via the Micro–Kjeldahl method (Naqvi et al. 2024) as follows: ~1 g of the sample was mixed with 0.2 g of copper sulfate, 3 g of potassium sulfate, and 10 mL of sulfuric acid in a digestion tube. The mixture was then placed in a digestion instrument and slowly heated to 420°C for 1 h until the liquid turned green and transparent. After cooling, the solution was distilled and titrated against the HCl standard solution. The formula for crude protein content was as follows:

(3) $$X=\frac{\left(V_{1}-V_{2}\right)\times c\times 0.0140}{m\times V_{3}/100}\times F\times 100$$

X represents the protein content of the sample (g/100 g); V_1_ represents the volume of sulfuric acid or hydrochloric acid standard titration solution consumed by the sample solution (mL); V_2_ represents the volume of sulfuric acid or hydrochloric acid standard titration solution consumed by blank reagent (mL); V_3_ represents the volume of the digestion solution (mL); C represents the concentration of the sulfuric acid or hydrochloric acid standard titration solution; m represents the mass of the sample (g); and F represents the nitrogen to protein conversion factor (generally 6.25 for most foods).

##### 2.4.1.6 Crude Fiber

Crude fiber content was determined via the method outlined by Handa et al. (2011) using AOAC 991.43 as follows: ~5 g of the dry sample was placed in a beaker with 1.25% H_2_SO_4_ and heated for 30 min to facilitate digestion. The mixture was then filtered, thoroughly washed with distilled water, and treated with 1.25% NaOH solution for an additional 30 min. The residue was filtered, dried overnight in an oven at 105°C, cooled in a desiccator for 1 h, and subsequently weighed to determine the crude fiber content. The formula for crude fiber content was as follows:

(4) $$X=\frac{\left(M_{2}-M_{1}\right)}{m}\times 100$$

X represents the dietary fiber content of the sample (%); m_1_ represents the mass of the crucible (g); m_2_ represents the mass of the crucible and residue (g); and m represents the mass of the sample (g).

##### 2.4.1.7 Carbohydrates

Carbohydrate content (A_1_) was calculated using the following formula:

(5) $$A_{1}=100-\left(A_{2}+A_{3}+A_{4}+A_{5}+A_{6}\right)$$

A_1_ represents the carbohydrate content (g/100 g); A_2_ represents the protein content (g/100 g); A_3_ represents the fat content (g/100 g); A_4_ represents the moisture content (g/100 g); A_5_ represents the ash content (g/100 g); and A_6_ represents the dietary fiber content (g/100 g).

#### 2.4.2 Tissue Structure Measurements

##### 2.4.2.1 Texture Profile Analysis

A PILOT texture analyzer was utilized for texture profile analysis (TPA) on the samples according to the method outlined by Manli Zhang et al. (2022), with only slight modifications. The samples were cut into cylindrical sections (1.5 × 1.5 × 2 cm) after removing the casings and then compressed to 60% for two consecutive cycles using a P/50 probe. The instrument parameters were set as follows: pre‐test speed, 2 mm/s; test speed, 1 mm/s; and post‐test speed, 2 mm/s. The tests were conducted in triplicate, and the average values for properties such as hardness, elasticity, adhesiveness, and chewability were recorded.

##### 2.4.2.2 Shear Force

A texture profile analyzer was used to determine the shear force of the samples via a modified method from Chen et al. (2010). The samples were cut into cylindrical sections (1.5 × 1.5 × 2 cm) after removing the casings, and then the shear force was measured perpendicular to the extrusion direction. The instrument parameters were set as follows: pre‐test speed, 2 mm/s; test speed, 1 mm/s; and post‐test speed, 2 mm/s. The tests were conducted in triplicate, and the average values were recorded.

##### 2.4.2.3 Total Express Juice Rate

The total express juice rate was determined by removing the casing from the sausage and sampling from different positions along the sausage body, based on a modified method from Pietrasik and Soladoye (2021) and Carballo et al. (1995). The sausage was cut into slices 0.5 cm thick, and a weight of 5 kg was applied to the slices for 2 min. The masses before and after pressing were measured and recorded for each sample. The tests were also conducted in triplicate, and the average values were calculated as follows:

(6) $$\text{Total pressure juice yield}=\frac{M-m}{M}\times 100$$

M represents the mass of the sample before pressing (g/100 g) and m represents the mass of the sample after pressing (g/100 g).

#### 2.4.3 Determination and Evaluation of Nutrient Components

##### 2.4.3.1 Amino Acid Determination

The amino acids present in the samples were determined using an automatic amino acid analyzer, based on a modified method from Golly (2019) as follows: ~50 mg of the sample was dissolved in 5 mL of 6 mol/L HCl solution, evaporated using a nitrogen blower for 20 s, and dried in a constant‐temperature blast oven at 110°C ± 2°C for 24 h. The solution was then diluted to 50 mL with water and filtered through a 0.45 μm filter membrane. Subsequently, the solution was extracted with 5 mL of hexane, shaken for 30 s, and allowed to stand for 20 min. Finally, 1 mL of the extract was diluted with 2 mL of distilled water and injected into the automatic amino acid analyzer.

##### 2.4.3.2 Amino Acid Composition Evaluation

Amino acid content is critical to the nutritional value of food. The specific calculation method was based on the essential amino acid requirement model stipulated by FAO/WHO.

#### 2.4.4 Functional Characteristic Analysis

##### 2.4.4.1 Water‐Holding Capacity (WHC) and Oil‐Holding Capacity (OHC)

With modifications based on the work of Ketnawa and Rawdkuen (2023) the stripped meat analogue samples were cut into cubic sections (10 × 10 × 10 mm) and weighed (W_0_/O_0_). The cubes were then placed in pre‐weighed 15 mL centrifuge tubes containing 5 mL deionized (DI) water (W_1_/O_1_), left to stand at room temperature for 5 min, and centrifuged at 4°C, 3500 rpm for 30 min. The supernatant was discarded, and the tubes with the residues were inverted, left to stand for 5 min, and then reweighed (W_2_/O_2_). The OHC determination method was the same as the WHC method, with walnut oil replacing distilled water. WHC and OHC were calculated using Equations (7) and (8), respectively:

(7) $$\mathrm{WHC}=\frac{W_{2}-W_{1}-W_{0}}{W_{0}}$$

(8) $$\mathrm{OHC}=\frac{O_{2}-O_{1}-O_{0}}{O_{0}}$$

W_2_/O_2_ = volume of the sample after centrifugation (g/100 g); W_1_/O_1_ = volume of the centrifuge tube (g/100 g); and W_0_/O_0_ = weight of the sample (g/100 g).

##### 2.4.4.2 Expansion Force (WSC)

Expansion force tests were conducted via a modified method based on the work of Sowbhagya et al. (2007). The tests were conducted as follows: 2 g of the sample was placed in 25 mL of distilled water for 24 h, and the volume of the precipitate in a graduated cylinder was recorded. The formula for WSC was as follows:

(9) $$\mathrm{WSC}=\frac{V_{1}-V_{0}}{W_{0}}$$

*V*_1_ represents the volume after hydration (g/100 g), *V*_0_ represents the volume before hydration (g/100 g), and W_0_ represents the weight of the sample (g/100 g).

#### 2.4.5 Biomimetic Sensor Evaluation

##### 2.4.5.1 Electronic Nose Evaluation

The flavor of the meat analogue was evaluated using a PEN3 portable electronic nose system. The system comprised a 10‐metal‐oxide‐thick‐film gas sensor array labeled as (W1C), (W5S), (W3C), (W6S), (W5S), (W1S), (W1W), (W2S), (W2W), and (W3S). The operational parameters for the electronic nose were set as follows (Li, Yang, et al. 2023): Pre‐sampling time, 5 s; measurement time, 90 s; wash time, 60 s; and flow rate, 400 mL/min for both the sample and carrier gas (filtered dry air) in non‐dilution mode. Data collection was performed between 46 s and 50 s, with the response of each sensor reported as G/G0, where G and G0 represent the resistance values of the sensor exposed to sample volatiles and clean air, respectively.

##### 2.4.5.2 Electronic Tongue Evaluation

The texture characteristics of the meat analogue were evaluated using an SA‐402B electronic tongue, equipped with a sensor array, data acquisition system, and analysis system. The analysis system comprised five taste analysis sensors (C00, AE1, CA0, CT0, and AAE) and two reference electrodes. The operational parameters for the electronic tongue were set as follows (Huang et al. 2023): homogeneous mixing ratio of 1:10 sample with pure water, followed by centrifugal filtration of the supernatant for detection. The cleaning times were set at 90, 120, and 120 s for the cleaning solution, while the conditioning time was 30 s and the testing time was 30 s. The initial taste value was output after cleaning with the reference solution for 3 s; the sensors were then inserted into a new reference solution for 30 s for taste recovery. The five taste sensors (C00, AE1, CA0, CT0, and AAE) were tested four times, with the average of the last three cycles recorded as the test result. Tables 4 and 5 presents the sensor information.

**TABLE 4:** Sensor information for electronic tongue evaluation.

Transducers	Basic taste	Taste information	
		Preference	Ponder over
C00	Acidic and bitter	Bitterness	Aftertaste‐B
AE1	Astringent	Astringency	Aftertaste‐A
CA0	Sourness	Sourness	×
CT0	Salty	Saltness	×
AAE	Freshness	Umami	Richness

**TABLE 5:** Basic composition of different plant protein sources and their water‐holding capacity.

Plant protein	Moisture (%)	Dietary fiber (g/100 g)	Crude protein (g/100 g)	Crude fat (g/100 g)	Water holding capacity (%)
WVS	9.12 ± 0.22a	13.68 ± 0.15a	84.9 ± 0.36a	1.41 ± 0.92c	726.67 ± 207.44a
SBMI	7.24 ± 0.23c	9.73 ± 0.18b	83.6 ± 0.57a	18.71 ± 0.37a	226.67 ± 32.15c
VGMS	7.78 ± 0.16b	4.13 ± 0.12c	71.7 ± 0.28b	5.99 ± 0.00b	506.67 ± 57.74b

*Note:* Values with different letters within one row are significantly different (*p* < 0.05).

#### 2.4.6 Sensory Evaluation

Ten food science professionals trained in sensory evaluation assessed the meat analogue based on its appearance, texture, mouthfeel, flavor, and overall acceptability.

### 2.5 Data Processing and Statistical Analysis

Data were processed and analyzed using SPSS 26.0 and Origin 2024 software. RCO was designed using the RCOPTNS software program. All experiments were conducted in triplicate and results were expressed as mean ± standard deviation. A *p*‐value > 0.05 indicated no significant difference, while a *p*‐value < 0.05 indicated a significant difference.

## 3 Results and Analysis

### 3.1 Analysis of the Basic Composition of Different Plant Protein Sources and Their Water Holding Capacity

The characteristics of raw materials determine the subsequent processing and product performance of plant protein sausage. Table 4 presents the results of the composition analysis (moisture, dietary fiber, protein, fat content, and water holding capacity) for walnut meal, soybean isolate protein, and gluten. The moisture content of walnut meal was 9.12%, which was significantly higher than that of soybean isolate protein (7.24%) and gluten (7.78%). Significant differences were observed between the other two groups, which might be associated with the inherent properties of the raw material. Furthermore, the total dietary fiber content of walnut meal was 13.68/100 g, and its protein and crude fat content were 84.9/100 g and 1.41/100 g, respectively. These values indicated that the walnut meal had high protein content and low fat content. In addition, the water‐holding capacity was 726.67%, which was three times and 1.4 times that of soybean isolate protein and gluten, respectively. These findings verified the excellent quality of walnut meal extrudates. This confirmation provided a scientific basis for the research and development of new protein meat products Table 5.

### 3.2 Establishment of Fuzzy Matrix Results

According to the sensory evaluation results, one person rated the appearance of the first group of samples as excellent, six rated it as very good, three rated it as fair, and none rated it as poor. Therefore, T1 = {0.2, 0.5, 0.3, 0}. Following this pattern, the summarized data were input into the fuzzy matrix model to obtain the results:

$$R_{1}=\left|\begin{array}{cccc} 0.2 & 0.5 & 0.3 & 0\\ 0.3 & 0.5 & 0.2 & 0\\ 0 & 0.2 & 0.4 & 0.4\\ 0 & 0.1 & 0.4 & 0.5 \end{array}\right|$$

Ssimilar results were obtained for *R2–R33*.

According to the principles of fuzzy mathematics and matrix transformation, the evaluation result of each group L = Fuzzy Matrix *R* × Weight Matrix AU, that is, *L = R × AU*, gives the fuzzy mathematical comprehensive sensory evaluation value. This approach avoids discrepancies that may arise from using maximum and minimum algorithms. The weights for the walnut protein sausage additives were defined as X = {0.26, 0.26, 0.22, 0.26}. Applying the fuzzy principle *L = R × AU* gives the sensory evaluation results for the walnut protein sausage. *L*_*1*_ *= AU × R* = {0.26, 0.26, 0.22, 0.26} ×.

$$\left|\begin{array}{cccc} 0.2 & 0.5 & 0.3 & 0\\ 0.3 & 0.5 & 0.2 & 0\\ 0 & 0.2 & 0.4 & 0.4\\ 0 & 0.1 & 0.4 & 0.5 \end{array}\right|$$

= {0.13, 0.33, 0.32, 0.22}. Similarly, we obtain *L*_*2*_ = {0.08, 0.38, 0.42, 0.12} and *L*_*33*_ = {0.53, 0.42, 0.05, 0.00}. The fuzzy comprehensive evaluation score was calculated as *W = K × L*, where the evaluation grades *K* = {96, 85, 74, 50}, and *L*_*1*_ = {12.60, 28.19, 23.77, 10.79}. Thus, the fuzzy comprehensive score for walnut protein sausage is *W*_*1*_ = *K × L*_*1*_ = {12.60, 28.19, 23.77, 10.79} × {96, 85, 74, 50} = 75.36. Similarly, we obtain *W*_*2*_ = 76.98 and *W*_*33*_ = 90.32.

### 3.3 Optimization Condition Design and Results of RCO for Walnut Protein Sausage

#### 3.3.1 First Round of RCO Experiment

The optimized factors for salt, sugar, modified starch, egg white powder, walnut oil, lab‐prepared spices, and walnut tissue protein were input into the RCO program. The 14 groups of random cyclic search scoring values obtained were further input into the system for centroid search experiments, which resulted in 4 additional groups; which in turn led to a total of 18 groups. Table 6 presents the results from the first round of experiments.

**TABLE 6:** First round of RCO experiments.

Number of cycles	Experiment number	Salt	Sugar	Modified starch	Powdered egg white	Walnut oil	Spice blend	Walnut tissue protein	Sensory evaluation
First round cyclic randomized search design	1	4.8	3.9	8.4	1.8	1.7	1.7	43.7	77.58
	2	1.9	4.4	5.2	4	1.9	1	81.8	76.98
	3	4.5	4.1	9.7	4.5	1.3	1.3	40.9	75.63
	4	1.8	5	8.6	4.9	1.6	1.1	63.7	78.42
	5	4.7	1.2	6.7	3.8	1.3	1.7	58.7	76.84
	6	1	2.4	7.6	7.1	1.1	1.6	87	82.03
	7	3	4	7.6	2.2	1.6	0.7	88.8	82.31
	8	2.1	4.1	7.5	1.2	1	1.4	70.1	81.38
	9	1.9	4.9	7.9	6	2	0.6	98.4	82.65
	10	1.4	1.6	6.5	3.6	1.5	1.9	44.5	81.38
	11	2.9	1.2	5.8	3.9	1.2	1.7	52.4	76.76
	12	2.6	3.5	6.5	6.7	1.5	0.6	42.3	80.07
	13	4.4	4.7	9.4	3.5	1.2	1.1	72.7	79.79
	14	2.1	2.1	5.7	4.1	1.3	1.5	81.4	75.61
Centre of gravity search	15	2.4	4.1	7.5	3.6	1.5	1.1	73	76.15
	16	2.7	3.7	7.4	4	1.6	1.2	71.4	75.56
	17	2.8	3.6	7.4	3.3	1.4	1.3	68.6	78.03
	18	2.6	3.7	7.4	3.9	1.5	1.3	68.7	77.39

**FIGURE 1:**
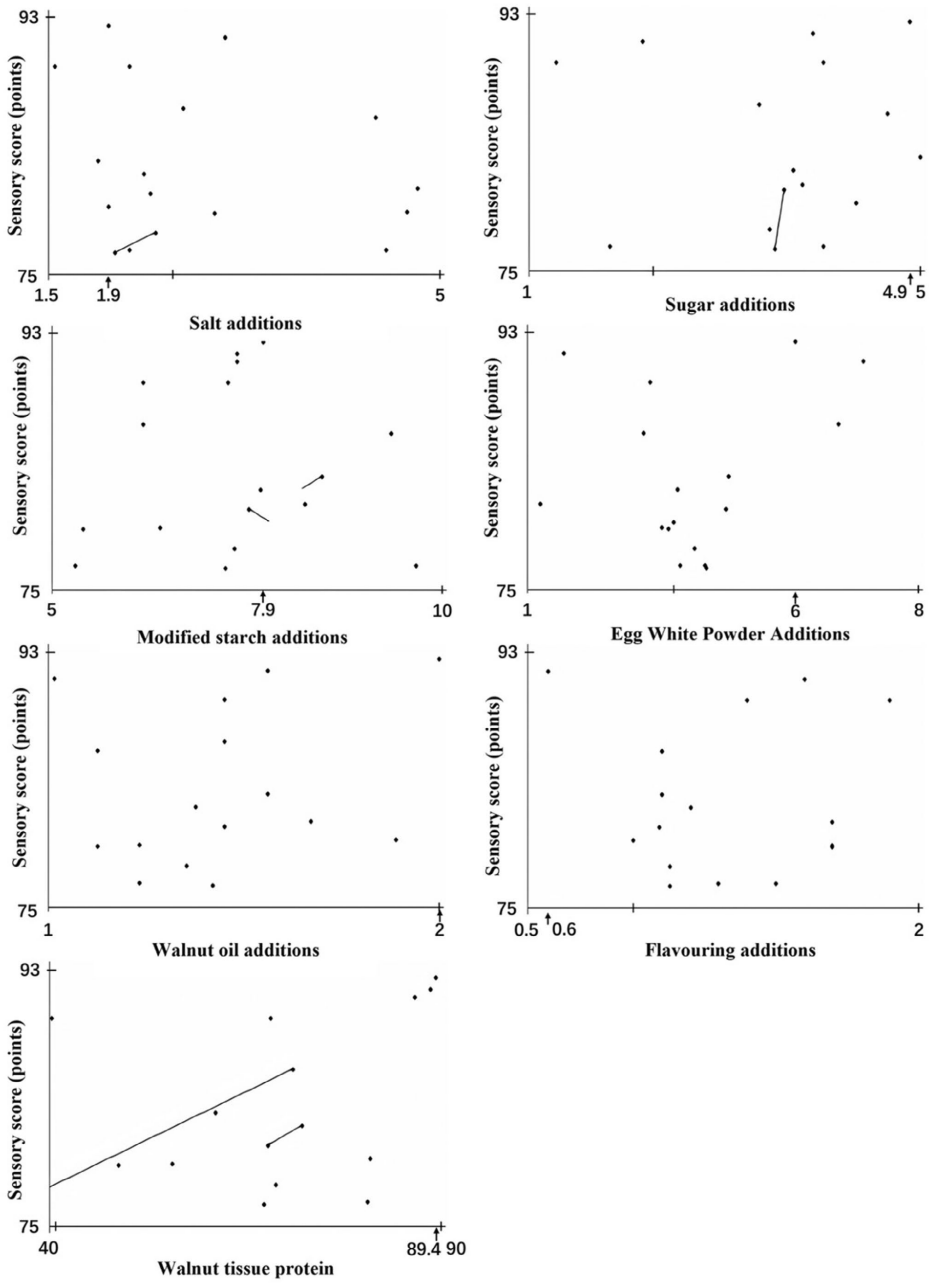
First round conditional mapping diagram.

Mapping optimisation diagrams were plotted based on the results from the first round of RCO experiments. Figure 1 shows the mapping optimisation diagrams for the first round of experiments. Each factor corresponds to an optimal mapping value for the sensory score on the horizontal axis (Figure 1). The first round optimisation results were as follows: 1.9% salt, 4.9% sugar, 7.9% modified starch, 6.0% powdered egg white, 2.0% walnut oil, 0.6% lab‐prepared spices, and 98.4% walnut tissue protein. However, the points on the mapping diagrams were relatively scattered, and the directions of the lines and curves were unclear. This observation indicated that the first round of experiments did not effectively reflect the optimisation results. Therefore, a second round of cyclic experiments was required.

#### 3.3.2 Second Round of RCO Experiment

Considering the results from the first round of cyclic experiments, the second round of experiments, which comprised 11 groups of random search experiments and 4 groups of centroid experiments, was conducted. Table 7 presents the experimental design and results of the second round of experiments.

**TABLE 7:** Second round of RCO experiments.

Number of cycles	Experiment number	Salt	Sugar	Modified starch	Powdered egg white	Walnut oil	Spice blend	Walnut tissue protein	Sensory evaluation
Second round cyclic randomized search design	19	1.6	4	5.6	6.5	1.9	0.7	60.3	79.48
	20	1.5	3.8	6.3	6.8	1.1	0.6	40.3	81.20
	21	1.9	1.6	5.9	4.6	1.5	0.9	63.5	81.70
	22	1.6	3.2	8.2	4.4	1.3	0.5	80.7	78.31
	23	1.5	5.1	5.4	6.4	2.3	0.5	55.6	72.65
	24	1.9	1.6	6.8	4.2	1.8	1	80.9	86.20
	25	1.6	5.3	8.4	5.7	1.9	0.5	41.7	85.39
	26	1.7	2.2	5.9	5.7	2.5	0.8	85.8	79.06
	27	2	3.6	8.6	4.7	1.9	0.9	87.3	83.09
	28	1.9	5.2	7	5.5	2.3	0.7	59.2	75.93
	29	1.7	5.3	7.7	4.1	1.1	0.8	43.5	82.87
Centre of gravity search	30	1.7	3.6	7	5.2	1.6	0.8	59.6	84.2
	31	1.8	3.3	7.1	5.1	1.7	0.8	63.3	85.01
	32	1.8	3.4	7	5.1	1.8	0.8	66.1	92.01
	33	1.7	3.7	7	5.4	1.7	0.8	62.8	90.31

**FIGURE 2:**
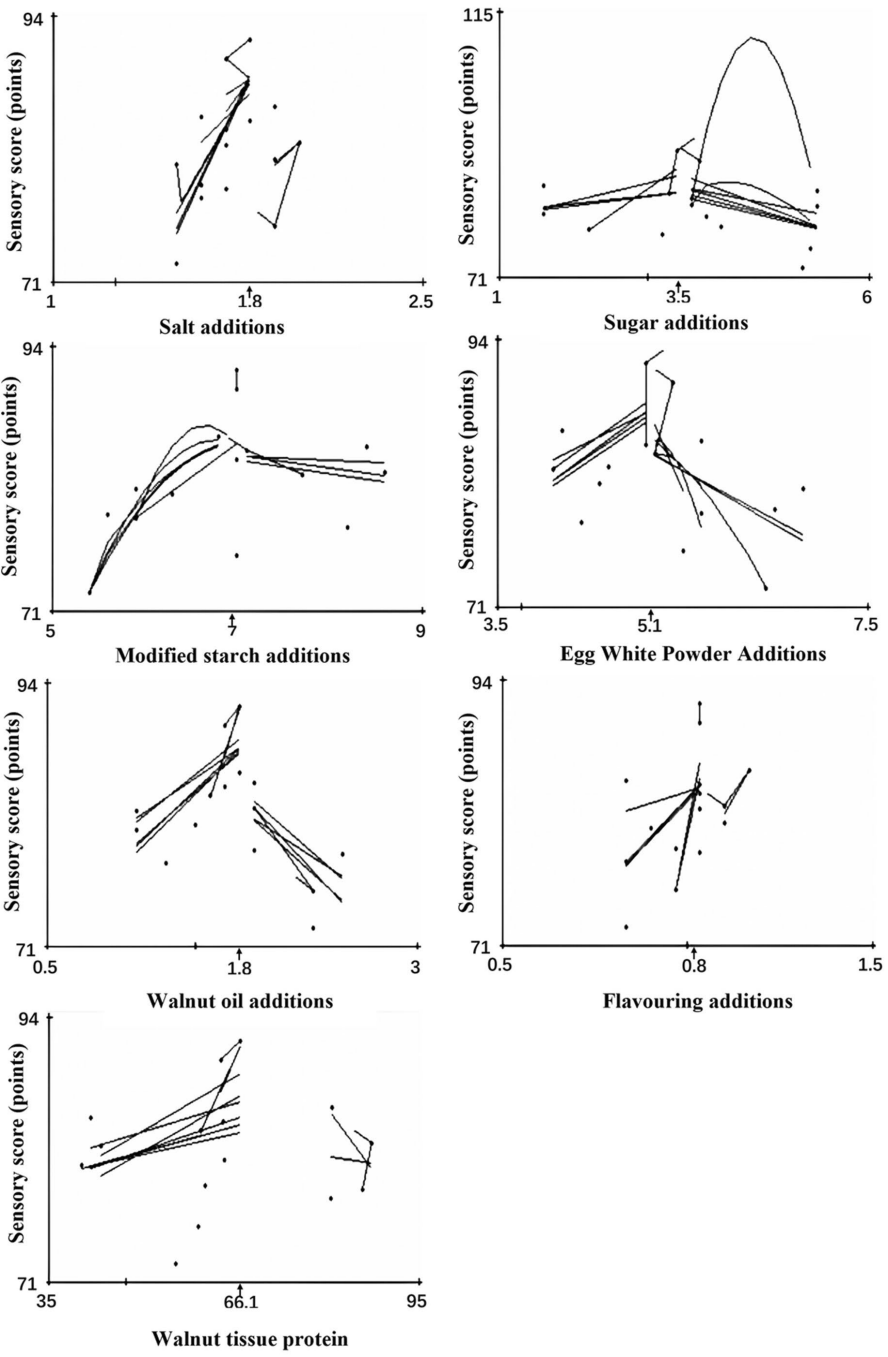
Second round conditional mapping diagram.

The results indicated that the sensory score for walnut protein sausage in the second round of experiments was higher than that in the first round of experiments (Figure 2). The lines and curves in the diagrams converged towards the optimal values for each factor, indicated by the arrows on the horizontal axis. The optimized process parameters were as follows: 1.8% salt, 3.4% sugar, 7% modified starch, 5.1% egg white powder, 1.8% walnut oil, 0.8% lab‐prepared spices, and 66.1% walnut tissue protein.

#### 3.3.3 Verification Experiment

The optimized process parameters (1.8% salt, 3.4% sugar, 7% modified starch, 5.1% egg white powder, 0.8% lab‐prepared spices, and 66.1% walnut tissue protein) were used for the preparation of walnut protein sausage. The prepared sausage achieved a sensory evaluation score of 92.7 points; this was significantly higher than that (92.01 points) achieved by the sausage prepared using the optimal process parameters from the first round of experiments. These findings verified the accuracy and reliability of the RCO results.

### 3.4 Determination of the Basic Physicochemical Indices of Different Plant‐Based Protein Sausages

#### 3.4.1 Determination of the Basic Components of Different Plant‐Based Protein Sausages

The color, moisture, crude fat, protein, dietary fiber, and carbohydrate content of walnut protein sausage, soybean protein sausage, wheat gluten protein sausage, and commercially available plant‐based protein sausage were analyzed and determined (Table 8).

**TABLE 8:** Basic components in different plant‐based protein sausages.

Sample number	WVS	SBMI	WGMS	VBPI
L*	44.39 ± 3.25^c^	45.96 ± 1.87^c^	59.01 ± 1.32^a^	51.98 ± 0.77^b^
a*	6.66 ± 0.63^c^	7.55 ± 0.64^c^	8.76 ± 0.83^b^	15.24 ± 0.17^a^
b*	6.14 ± 1.10^a^	7.76 ± 0.48^a^	13.47 ± 5.14^b^	22.91 ± 0.32^a^
Moisture (%)	46.10 ± 0.02^b^	43.90 ± 0.04^bc^	45.40 ± 0.00^c^	54.75 ± 0.38^a^
Ash content (g/100 g)	4.00 ± 0.00^a^	3.33 ± 0.00^ab^	2.40 ± 0.00^ad^	2.80 ± 0.00^ac^
Crude fat (g/100 g)	6.59 ± 0.325^bc^	7.4 ± 0.189^b^	7.73 ± 0.193^b^	9.95 ± 0.003^a^
Crude fat (g/100 g)	20.65 ± 0.07^c^	24.10 ± 0.14^b^	27.20 ± 0.14^a^	12.85 ± 0.07^d^
Dietary fiber (g/100 g)	7.40 ± 0.02^a^	5.75 ± 0.03^c^	6.44 ± 0.04^b^	3.65 ± 0.03^d^
Dietary fiber (%)	15.26 ± 0.00	15.52 ± 0.00	10.83 ± 0.00	16.00 ± 0.00

*Note:* According to Duncan's test, mean values with different superscripts in the same column are significantly different (*p* < 0.05). Values are represented as mean ± standard deviation. (1) WVS (walnut protein sausage); (2) SBMI (soybean protein sausage); (3) WGMS (wheat gluten protein sausage); (4) VBPI (commercial vegetarian meat sausage).

Color is a critical factor in the sensory evaluation of food quality (Xu et al. 2022). Significant color differences were observed among the different plant‐based protein sausages (*p* < 0.05). The brightness of the WGMS samples was notably high (L* = 59.01), which was attributed to the inherent color of the raw materials and the processing methods used. Plant‐derived red pigments were added during preparation to simulate the appearance of red meat. These additives affected the overall color of the product and reduced the impact of raw materials on the final color of the product. The color of the WGMS samples was closest to that of commercial products, while the brightness of the WVS samples was similar to that of the SBMI samples. Overall, no significant differences in color were observed among the protein sausages prepared from different raw material ratios (*p* < 0.05). All sausages exhibited colors similar to that of commercial products. The moisture content of the protein sausages ranged from 43.90% to 54.75%. The ash content ranged from 2.40 to 4.00 g/100 g, with the highest ash content exhibited by WVS samples and the lowest by WGMS samples. The crude fat content ranged from 6.59 to 9.95 g/100 g, with the lowest crude fat content exhibited by WVS samples and the highest by VBPI samples. These differences were attributed to the varying characteristics of the raw materials and the processing methods used. Fewer fat analogues were added during the preparation of WVS, which resulted in a lower fat content compared with commercial sausages. The crude protein content ranged from 12.85 to 27.20/100 g, and VBPI exhibited the lowest crude protein content, which significantly differed from that of the other three protein sausages. This observation indicated that the homemade protein sausages contained high‐quality protein. Among the four sausage samples, WVS exhibited the highest dietary fiber content at 7.40/100 g, followed by WGMS at 6.44/100 g. Zhu et al. (2021) reported that dietary fiber exhibited excellent fat adsorption capabilities, which indicated that higher dietary fiber content correlates with lower fat content. This finding was consistent with the findings of previous research. Carbohydrates, mainly composed of polysaccharides and monosaccharides, are the major nutrients required for energy by the human body (Iizuka 2021). The carbohydrate content of the four sausage samples ranged from 10.83% to 16.00%. VBPI samples exhibited the highest carbohydrate content and WGMS samples exhibited the lowest content. The high carbohydrate content was attributed to the presence of non‐fiber carbohydrates, such as sugars, semi‐fibers, structural polysaccharides, β‐glucans, chitin, and pectin (Kalač 2013).

### 3.5 Determination of the Tissue Structure of the Different Plant‐Based Protein Sausages

WVS, SBMI, and VBPI samples exhibited similar cohesiveness, which indicated a similar texture among the samples (Ma et al. 2024) (Table 9). Elasticity, adhesiveness, chewiness, and hardness are the main parameters that characterize texture. Significant differences (*p* < 0.05) were observed in the elasticity, adhesiveness, chewiness, and hardness of structural proteins derived from different sources. The VBPI samples exhibited the highest elasticity and adhesiveness, with contents of 12.09% and 18.63%, respectively. This result indicated that VBPI exhibited good elasticity and adhesiveness. Following VBPI, WGMS samples exhibited values of 6.5% for elasticity and 5.98% for adhesiveness. This was mainly due to the extrusion of wheat gluten protein from gluten flour, which contained a substantial amount of glutenin protein (Chen et al. 2021). Wheat gluten protein consists of glutenin and gliadin. During extrusion, gliadin forms a network structure through intermolecular disulphide bonds and non‐covalent interactions, which creates continuous bands in the presence of water that results in enhanced viscoelastic properties (Barak et al. 2014). Chewiness is the energy required to chew a solid sample into a state suitable for swallowing; a lower chewiness value indicates a better texture (Wójtowicz et al. 2019). The SBMI samples exhibited the lowest chewiness at 6.49 mJ, followed by the WVS samples at 10.30 mJ (Table 8). This observation demonstrated that SBMI and WVS had better chewiness compared to the other two products. WVS samples exhibited low hardness (6.84 N), which might be attributed to high dietary fiber content. A loose dietary fiber network structure increases the volume‐to‐surface area ratio, which leads to a high content of soluble substances and a soft structure, which in turn results in low hardness (Xu et al. 2023). In contrast, commercial plant‐based protein sausages have low dietary fiber content and a relatively compact structure, which results in high hardness (16.46 N) (Li, Yang, et al. 2023). Additionally, WVS and SBMI samples exhibited higher total expressible juice rates compared with WGMS and VBPI samples. This observation indicated that WVS and SBMI sausages had good juice retention and a stable tissue structure.

**TABLE 9:** Texture of different plant‐based protein sausages.

Samples	Cohesiveness (ratio)	Elasticity (%)	Adhesiveness (N)	Chewiness (mJ)	Hardness (N)	Overall press‐out rate (g)
WVS	0.28 ± 0.02^b^	3.56 ± 0.17^c^	2.91 ± 0.58^c^	10.30 ± 1.77^bc^	6.84 ± 0.36^b^	7.30 ± 0.65^ab^
SBMI	0.28 ± 0.55^b^	1.96 ± 0.26^c^	3.39 ± 1.18^c^	6.49 ± 1.97^c^	7.95 ± 0.89^a^	7.61 ± 0.56^a^
WGMS	0.48 ± 0.02^a^	6.50 ± 0.43^b^	5.98 ± 0.02^b^	37.16 ± 0.04^b^	9.47 ± 0.01^a^	6.13 ± 0.74^a^
VBPI	0.28 ± 0.02^b^	12.09 ± 1.89^a^	18.63 ± 1.57^a^	165.94 ± 12.14^a^	16.46 ± 0.02^c^	5.08 ± 0.13^b^

*Note:* Values with different letters within one row are significantly different (*p* < 0.05).

### 3.6 Nutrient Composition Determination and Evaluation of Different Plant‐Based Protein Sausages

#### 3.6.1 Amino Acid Composition Determination and Evaluation of Different Plant‐Based Protein Sausages

Amino acid composition determines the nutritional value of proteins (Mao et al. 2014). The plant‐based protein sausages contained 16 amino acids, which included six essential amino acids (EAA) (lysine, phenylalanine, threonine, isoleucine, leucine, and valine) and 10 non‐essential amino acids (NEAA) (glycine, alanine, proline, tyrosine, serine, cysteine, histidine, arginine, glutamine, and aspartic acid) (Table 10). Among the 16 amino acids, glutamic acid (Glu), with a content range of 2.15–9.28 g/100 g, had the highest amino acid content and accounted for ~19% of total amino acid content. Glu is the main amino acid responsible for the umami intensity (Yang et al. 2020).

**TABLE 10:** Amino acid composition and content of different plant‐based protein sausages.

Different vegetarian sausages amino acid	WVS	SBMI	WGMS	VBPI
EAA				
Lys	1.13 ± 0.01^b^	1.43 ± 0.00^a^	0.49 ± 0.01^d^	0.72 ± 0.03^c^
Phe	0.97 ± 0.04^b^	1.21 ± 0.00^a^	1.32 ± 0.08^a^	0.70 ± 0.04^c^
Thr	0.68 ± 0.01^b^	0.83 ± 0.00^a^	0.66 ± 0.01^b^	0.46 ± 0.01^c^
Iso	0.87 ± 0.02^c^	1.08 ± 0.00^a^	0.97 ± 0.04^b^	0.60 ± 0.04^d^
Leu	1.48 ± 0.04^b^	1.84 ± 0.01^a^	1.84 ± 0.06^a^	0.99 ± 0.04^c^
Val	0.92 ± 0.01^b^	1.14 ± 0.01^a^	1.07 ± 0.04^a^	0.68 ± 0.04^c^
NEAA				
Gly	0.84 ± 0.08^a^	1.01 ± 0.09^a^	1.03 ± 0.09^a^	0.47 ± 0.01^b^
Ala	0.85 ± 0.03^b^	1.06 ± 0.02^a^	0.76 ± 0.03^c^	0.56 ± 0.01^d^
Pro	1.11 ± 0.01^b^	1.23 ± 0.07^b^	3.22 ± 0.11^a^	0.61 ± 0.03^c^
Tyr	0.43 ± 0.04^b^	0.57 ± 0.01^a^	0.62 ± 0.08^a^	0.37 ± 0.02^b^
Ser	0.79 ± 0.02^c^	0.98 ± 0.06^b^	1.15 ± 0.04^a^	0.61 ± 0.00^d^
Met	0.14 ± 0.01^c^	0.23 ± 0.01^b^	0.33 ± 0.01^a^	0.22 ± 0.01^b^
His	0.48 ± 0.01^d^	0.59 ± 0.00^a^	0.56 ± 0.01^b^	0.30 ± 0.01^c^
Arg	1.30 ± 0.02^b^	1.61 ± 0.03^a^	0.94 ± 0.01^c^	0.73 ± 0.02^d^
Glu	3.74 ± 0.08^b^	4.24 ± 0.06^b^	9.28 ± 0.31^a^	2.15 ± 0.14^c^
Asp	2.00 ± 0.03^b^	2.57 ± 0.01^a^	0.92 ± 0.01^d^	1.24 ± 0.04^c^
TAA	17.73	21.62	25.15	11.41
EAA	6.05	7.53	6.35	4.15
NEAA	11.68	14.09	18.8	7.26
F	7.39	8.88	11.98	4.42
EAA/TAA (%)	34	35	25	36
EAA/NEAA (%)	52	53	34	57
F/TAA (%)	42	41	48	39
CE/TAA (%)	10	10	6	9

*Note:* The total amino acid content is denoted as TAA; essential amino acids for the human body are denoted as EAA; non‐essential amino acids are denoted as NEAA; umami amino acids are denoted as F, which includes glutamic acid, aspartic acid, glycine, and alanine; the percentage of essential amino acids to total amino acids is denoted as EAA/TAA; the ratio of essential amino acids to non‐essential amino acids is denoted as EAA/NEAA; the percentage of umami amino acids to total amino acids is denoted as F/TAA. Values with different letters within one row are significantly different (*p* < 0.05).

EAA refers to amino acids that the human body cannot synthesize independently or produce at a rate sufficient to meet human needs and must be obtained from food. A prolonged deficiency of EAAs in the diet negatively impacts human health (Hou and Wu 2018). Lysine was the highest EAA content in plant‐based protein sausages and ranged from 0.49 to 1.43/100 g, followed by leucine, which ranged from 0.99 to 1.84/100 g (Table 9). The valine, threonine, phenylalanine, and isoleucine contents were similar. The total EAA content across the four plant‐based protein sausages ranged from 4.15 to 7.53/100 g, which accounted for 25%–36% of TAA. The total NEAA content ranged from 7.26 to 18.8/100 g, which accounted for 34% to 57% of TAA. The EAA/TAA ratios for the WVS, SBMI, and VBPI samples were 34%, 35%, and 36%, respectively. The EAA/NEAA ratios for these samples were 52%, 53%, and 57%, respectively. The ideal protein content recommended by the FAO/WHO (Kazuo 1995) indicated an EAA/TAA ratio of 35%–40% in high‐quality proteins and an EAA/NEAA ratio of 55%–60%. The plant‐based protein sausages prepared in this study exhibited protein content values similar to the recommended FAO/WHO values. This observation indicated the presence of high‐quality proteins in the prepared sausages (Vinayashree and Vasu 2021). WGMS exhibited a high umami amino acid content (11.98%), with glutamic acid as the predominant component at 9.28/100 g. This observation indicated that glutamic acid was the main component responsible for the flavor of WGMS. This finding was consistent with the findings of Vasconcelos.

### 3.7 Functional Characteristics of Different Plant‐Based Protein Sausages

**FIGURE 3:**
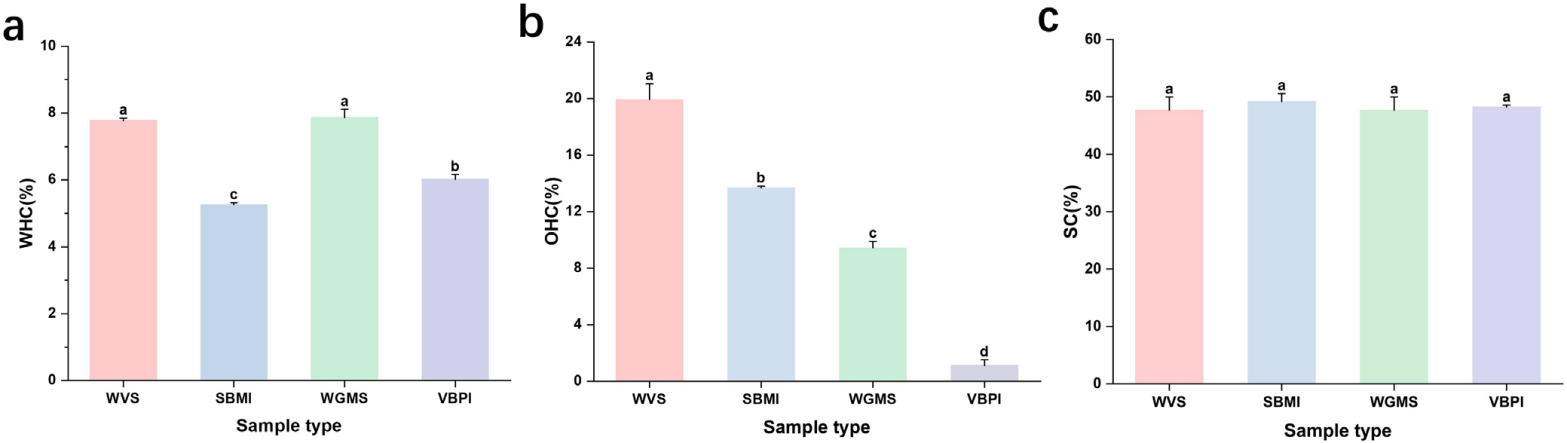
Functional characteristics of different plant‐based protein sausages. (a) water‐holding capacity (WHC); (b) oil‐holding capacity (OHC); and (c) swelling capacity.

Figure 3 shows the functional characteristics of various plant‐based protein sausages. These characteristics included WHC, OHC, and expansion capacity. The WHC values of the plant‐based protein sausages ranged from 5.26% to 7.87% (Figure 3a). WGMS exhibited the highest WHC at 7.87%, followed closely by WVS at 7.78%. The difference in the WHC of SBMI and VBPI compared with that of WGMS and WVS was significant (*p* < 0.05), which indicated that WVS and WGMS had superior WHC. The OHC values of the plant‐based protein sausages ranged from 1.1% to 19.92%, with significant differences observed (*p* < 0.05) (Figure 3b). WVS exhibited the highest OHC at 19.92%, followed by SBMI at 13.69%, WGMS at 9.43%, and VBPI at 1.11%. The variations in WHC and OHC could be attributed to the denaturation of proteins and the exposure of more hydrophobic sites owing to high‐temperature cooking (Osen et al. 2014). This process led to a more flexible protein conformation, which altered the WHC and OHC of the plant‐based protein sausages (Mozafarpour et al. 2019). The WHC and OHC of proteins increase with protein purity (Bao and Ertbjerg 2019). The expansion capacity values of the plant‐based protein sausages ranged from 47.69% to 49.69%, with no significant differences observed (*p* > 0.05) (Figure 3c).

### 3.8 Simulation Sensory Evaluation

#### 3.8.1 Analysis of Electronic Nose Results for Different Plant‐Based Protein Sausages

**FIGURE 4:**
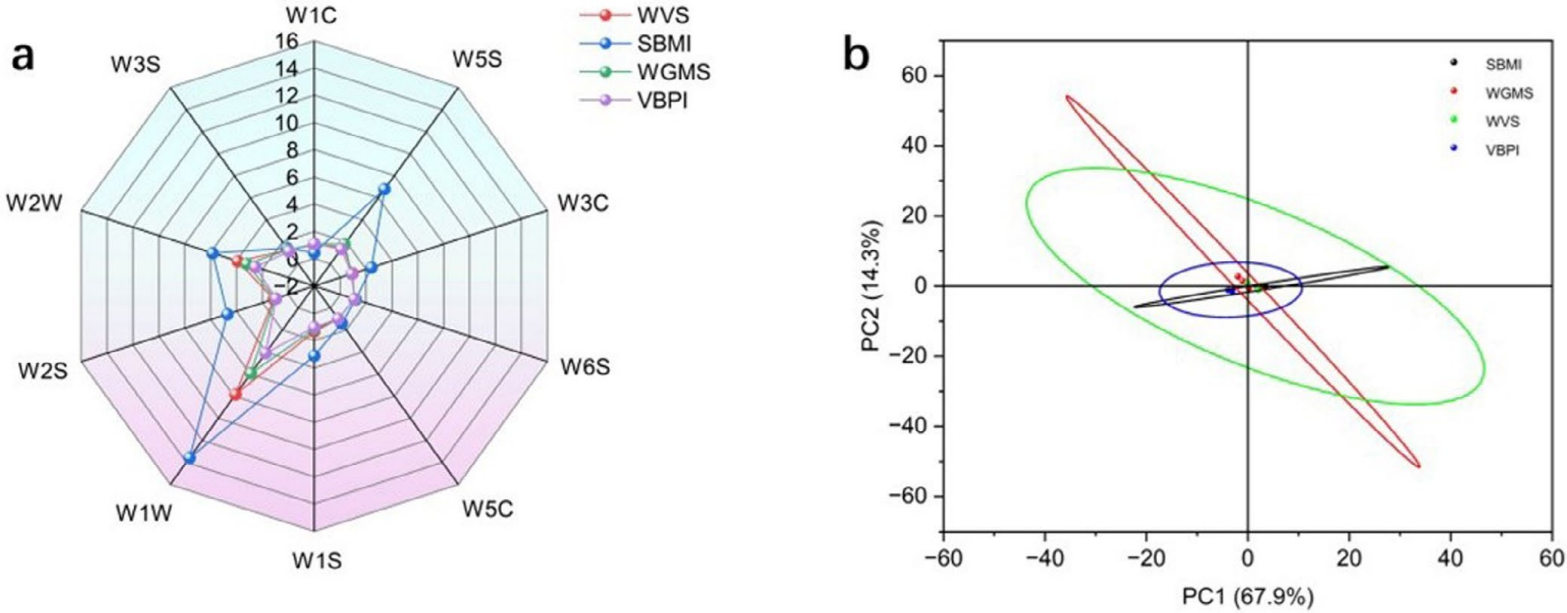
Radar and principal component analysis (PCA) plots for different plant‐based protein sausages using an electronic nose.

To visually represent the aroma characteristics of the different plant‐based sausage samples, radar charts were plotted based on the peak response intensity from 10 distinct electronic nose sensors. The response intensity of the sensors varied across the four plant‐based protein sausage samples, with notable differences observed mostly in the W2W, W1W, and W5S sensors (Figure 4a). The WVS, SBMI, WGMS, and VBPI samples exhibited high response values for the W1W, W2W, and W5S sensors, which indicated that these samples contained aromatic compounds, sulfides, and nitrogen compounds critical for distinguishing between them. The aromas of the four samples were similar, which also indicated similar profiles of aromatic compounds, sulfides, and nitrogen compounds. Notably, SBMI exhibited the highest response values, which indicated a greater concentration of these compounds. The contribution rates of the first principal component (PC1) and the second principal component (PC2) were 67.9% and 14.3%, respectively, with a total contribution rate of 82.2% for both components (Figure 4b). This observation indicated that PC1 and PC2 reflected the vast majority of information (> 80%) (Tang et al. 2021). The WVS, SBMI, WGMS, and commercial samples overlapped with each other, which indicated that the aroma characteristics of these four samples were similar.

#### 3.8.2 Analysis of Electronic Tongue Results for Different Plant‐Based Protein Sausages

The electronic tongue simulates the taste perception mechanism of living organisms by detecting changes in membrane potential generated through electrostatic or hydrophobic interactions between various taste substances and artificial lipid membranes. This technology facilitated the evaluation of five basic tastes (sour, sweet, bitter, salty, umami) and astringency. The electronic tongue was used to assess the sensory quality of WVS, SBMI, WGMS, and VBPI Table 11.

**FIGURE 5:**
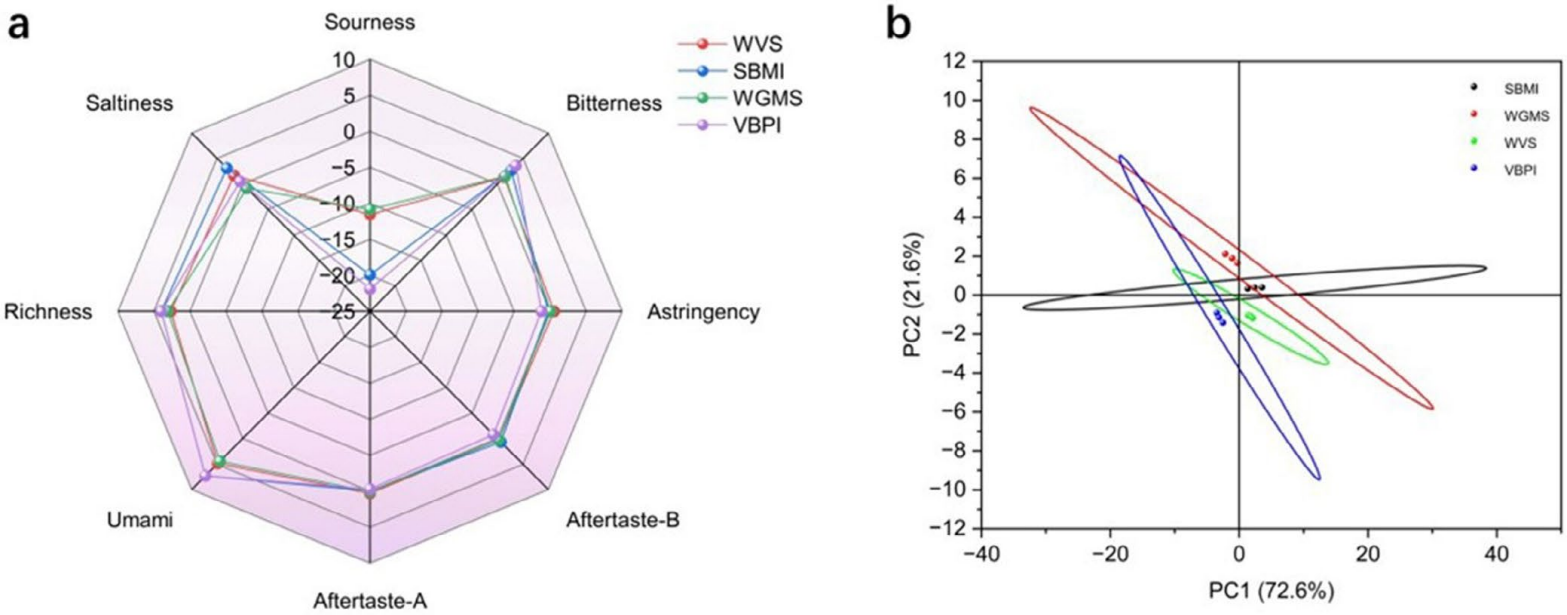
Radar and principal component analysis (PCA) plots of electronic tongue responses for different plant‐based protein sausages.

The different plant‐based protein sausage samples exhibited significant differences in effective evaluation indices for various tastes (*p* < 0.05) (Figure 5a). The response values for umami and umami aftertaste were greater than zero, while those for salty, bitter, astringent, astringent aftertaste, and bitter aftertaste were close to zero. The sour taste response value was less than zero. These observations indicated that the umami and umami aftertaste contributed to the flavor of the different plant‐based protein sausages, with notable contributions from umami and salty tastes. The analysis indicated that WGMS had the best salty taste, while VBPI had the best umami taste, with overlapping areas indicating similar flavor profiles among the samples.

PC1 (*X*‐axis) accounted for 72.6% of the total variance, while PC2 (Y‐axis) accounted for 21.6%, with a total contribution of 94.2% for both components (Figure 5b). This observation indicated that the PCA effectively reflected the majority of information about the samples. The variance contribution of PC1 was much greater than that of PC2, which indicated that larger distances between samples on the *X*‐axis corresponded to greater differences. Although notable separation was observed between samples on the *Y*‐axis, the smaller variance contribution of PC2 implied less actual difference. The relatively close distribution and overlapping areas of the samples further indicated similar flavor profiles.

### 3.9 Sensory Evaluation of Different Plant‐Based Protein Sausages

**FIGURE 6:**
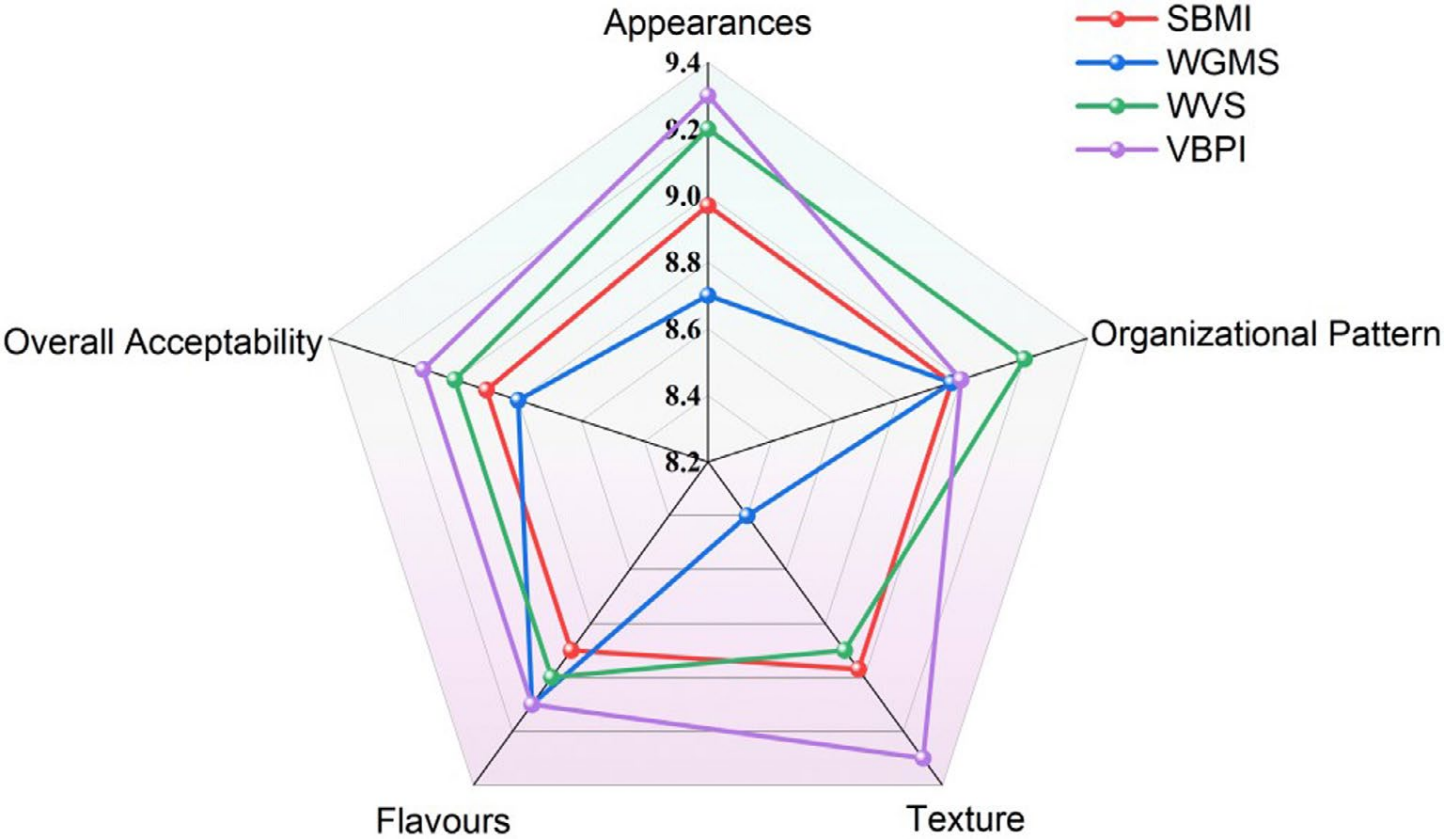
Radar plot of sensory evaluation results for different plant‐based protein sausages.

Sensory evaluation is a comprehensive assessment method, widely recognized in the food industry, that considers the appearance, texture, mouthfeel, flavor, and overall acceptance of samples. Figure 6 shows the evaluation results for the appearance, texture, mouthfeel, flavor, and overall acceptance of the four samples.

Sensory evaluation is essential for flavor analysis of food products. Using a flavor wheel to describe product characteristics facilitates intuitive comparisons between samples. A sensory panel evaluated five indicators—appearance, texture, mouthfeel, flavor, and overall acceptance—for the plant‐based protein sausages, and radar charts were plotted based on sensory scores for each sample. Significant differences in overall flavor intensity were observed among the different plant‐based protein sausages (Figure 6). VBPI exhibited the highest scores across all indicators. The VBPI samples exhibited a more intense meaty aroma than the other three samples. This observation was due to the addition of various aromatic seasonings during preparation, where the optimal release of aromatic components during cooking—achieved through precise temperature and time control—enhanced the pleasant aroma and mouthfeel of the plant‐based protein sausage. Additionally, WVS had the highest score for texture, which was consistent with texture analysis results. This was due to differences in raw materials and processing methods. Minimal variation was observed between the overall flavor profiles of the SBMI and WGMS samples. The scoring sequence was as follows for mouthfeel: VBPI (9.1 points) > WVS (9 points) > SBMI (8.9 points) > WGMS (8.7 points).

#### 3.9.1 Correlation Analysis for Various Quality Indicators

**FIGURE 7:**
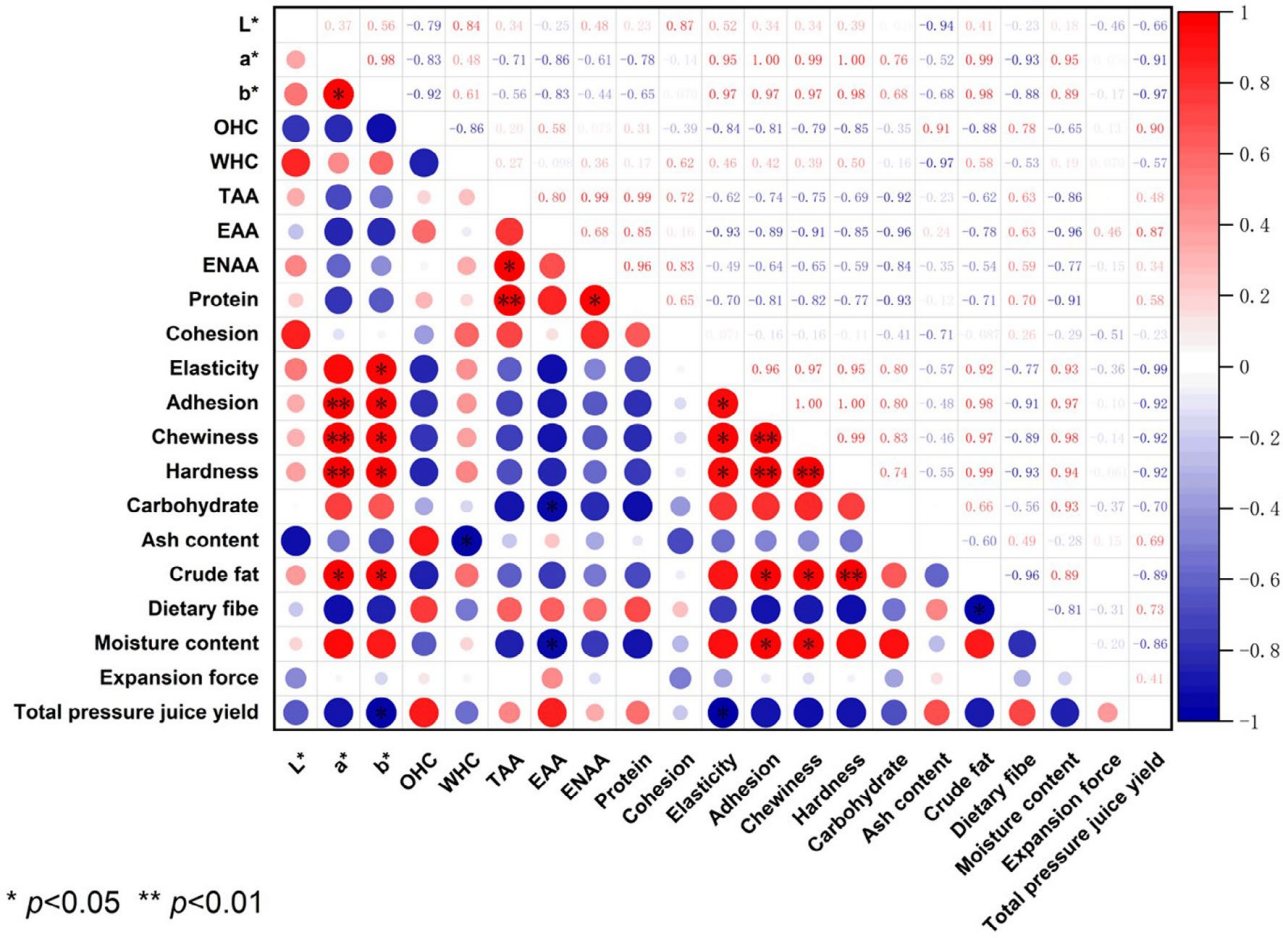
Correlation heat map between physicochemical properties and nutrients in the plant‐based protein sausages *n*. Highly significant correlation (*p* < 0.01); *Significant correlation (*p* < 0.05).

Pearson correlation analysis was conducted on various indicators to elucidate the relationships between the physicochemical properties, functional properties, and nutritional properties of different plant‐based protein sausages (Figure 7). The results indicated that most of the quality indicators for the plant‐based protein sausages exhibited significant or highly significant correlations. The a* value was highly positively correlated with gumminess, chewiness, and hardness, while the b* value was positively correlated with elasticity, gumminess, and chewiness, but negatively correlated with total expressible juice. Crude protein was highly positively correlated with total amino acids and positively correlated with NEAAs, which indicated a close relationship between crude protein and umami substances in plant‐based protein sausages. Crude fat exhibited a highly positive correlation with hardness and a negative correlation with total expressible juice. Studies suggest (Sato et al. 1999) that the interaction forces between fat molecules create a structural network that potentially increases the overall hardness of plant‐based protein sausages. As fat content increases, the total expressible juice correspondingly decreases. Moisture content was negatively correlated with crude protein content but positively correlated with gumminess and chewiness, which indicated that moisture effectively enhanced the gumminess and chewiness of the plant‐based protein sausages.

### 3.10 Evaluation of Economic Benefits

Walnut vegetarian meat was 58.1% lower in cost than pure soy vegetarian meat (Tables 12, 13). This difference was mainly attributed to the unit price of walnut meal (60% of the total) being only 8.8% that of soya isolate protein. Walnut meal was used as a low‐cost protein substitute for high‐cost raw material. The 52.6% reduction in cost for walnut protein sausage compared with commercially available vegetarian meat was attributed to the fact that commercially available vegetarian meat predominantly comprised soybean protein (still a high‐cost protein) and not walnut meal. In subsequent experiments, through technological improvement, if the proportion of walnut meal in the compound vegetarian meat could be increased to 70%, then the cost of a ton of the product would be further reduced to ~7000 yuan. Thus, based on this cost advantage, the end products would be priced 15%–20% lower than the commercially available vegetarian meat. Moreover, the “high‐fiber + low‐fat” content enhances the consumer appeal for the product. For example, the company marketing the product promotes it as “vegetarian meat for fitness people.”

**TABLE 11:** Comparison of electronic tongue response values for different plant‐based protein sausages.

Samples	WVS	SBMI	VGMS	VBPI
Sourness	−11.5 ± 2.06^a^	−19.93 ± 1.56^b^	−10.81 ± 0.73^a^	−21.92 ± 1.20^b^
Bitterness	1.33 ± 0.20^c^	2.70 ± 0.42^b^	1.48 ± 0.27^c^	3.60 ± 0.40^a^
Astringency	0.54 ± 0.18^a^	−0.25 ± 0.26^c^	0.07 ± 0.10^b^	−1.21 ± 0.01^d^
Aftertaste‐B	0.29 ± 0.18^b^	0.71 ± 0.04^a^	0.28 ± 0.05^b^	−0.79 ± 0.05^c^
Aftertaste‐A	0.28 ± 0.18^a^	−0.08 ± 0.11^b^	0.06 ± 0.05^b^	−0.30 ± 0.05^c^
Umami	4.86 ± 0.56^b^	7.33 ± 0.43^a^	4.44 ± 0.17^b^	7.31 ± 0.38^a^
Richness	2.70 ± 0.39^b^	3.99 ± 0.39^a^	3.12 ± 0.18^b^	3.96 ± 0.06^a^
Saltness	1.69 ± 0.13^b^	3.08 ± 0.30^a^	−0.80 ± 0.07^d^	0.49 ± 0.60^c^

*Note:* Values with different letters within one row are significantly different (*p* < 0.05).

**TABLE 12:** Analysis of raw material costs.

Ingredients	Specification/Status	Price range (yuan/tonne)
Walnut meal	Food grade (protein ≥ 80%)	1800–2000
Soya isolate protein	Food grade (protein ≥ 90%)	20,000–23,000
Gluten	Food grade (protein ≥ 85%)	10,300–11,000

**TABLE 13:** Comparative cost analysis of different vegetarian meat products.

Vegetarian meat types	Raw material composition	Cost per tonne ($)	Difference in cost compared with complex proteins	Cost variance ratio
Complex protein vegetarian meat	Walnut meal 60% + soybean isolate 25% + gluten 15%	8112.5	—	—
Pure soya vegetarian meat	Soya isolate protein 90%	19,350	+11237.5	+138.5%
Commercially available vegetarian meat	Soya split protein 90%	17,100	+8987.5	+110.8%

## 4 Conclusion

**FIGURE 8:**
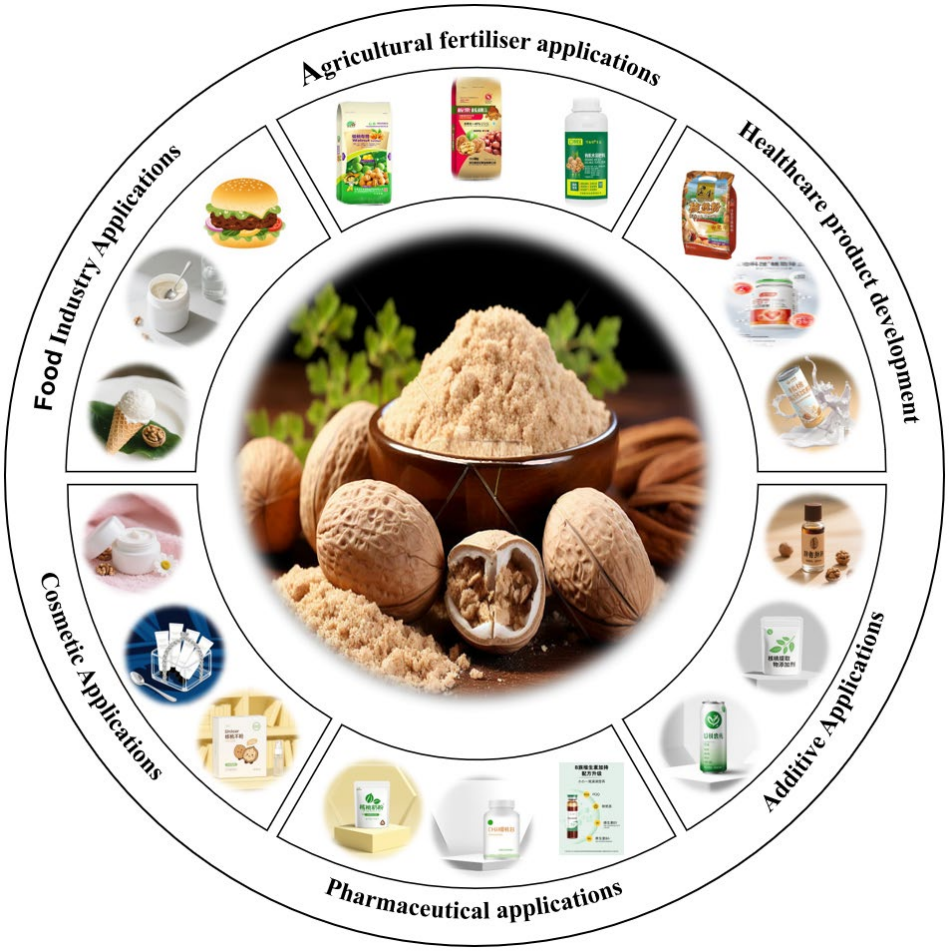
Future potential applications of walnut meal.

This study utilized fuzzy mathematics combined with RCO to investigate the effects of salt, sugar, modified starch, egg white powder, walnut oil, lab‐prepared spices, and walnut protein on sensory scores for walnut protein‐based sausage. A comprehensive evaluation system, based on the weights for appearance, texture, mouthfeel, and flavor, was used to determine the optimal formulation for walnut protein‐based sausages: 1.8% salt, 3.4% sugar, 7% modified starch, 5.1% egg white powder, 0.8% lab‐prepared spices, and 66.1% walnut protein. The sausages prepared using the optimal formula achieved a sensory score of 92.01. The optimized process for the preparation of walnut protein‐based sausages was accurate and feasible. The process provides a theoretical basis for future processing and production of walnut protein‐based sausages. According to multidimensional analysis of plant‐based protein sausages, the walnut protein‐based sausages had high dietary fiber content (7.40 g/100 g), low crude fat content (6.59 g/100 g), good WHC (7.78%), and good OHC (19.92%). These sausages also had a distinct walnut aroma, a smooth surface, and a uniform, full texture. Correlation analysis revealed a close relationship between crude protein, crude fat, moisture content, and the overall flavor of plant‐based protein sausages. The development of high‐quality walnut protein‐based sausages using walnut meal provides new resources and opportunities for the production of diversified plant‐based meat products, which in turn presents broad application prospects. However, further improvement of the walnut‐based product is required. Future research could focus on utilizing walnut by‐products, such as walnut meal, in combination with sensory and flavor enhancement techniques to develop a new range of walnut‐based protein products, which expands the application scope of walnut by‐products. To promote the use of walnut vegetarian meat for industrial applications, building a transformation path for product innovation, industry chain integration, and market cultivation is necessary. Specific recommendations are as follows: First, the development of a differentiated product matrix based on the distinctiveness of walnut meal nutritional characteristics. For the fitness crowd, the development of high‐protein walnut meal milk, cheese, and yogurt. For the middle‐aged and elderly market, the introduction of high‐fiber, low‐fat bread, cakes, biscuits, and other bakery products with walnut meal. For the leisure food industry, the development of extruded puffed protein bars and nut‐flavored jerky. Second, the entire industry chain should be optimized for synergistic cost reduction and efficiency (Figure 8). Walnut oil processing enterprises should establish a targeted cooperation mechanism to build a closed‐loop supply chain of “walnut meal recycling‐primary processing‐nutrient utilization” to reduce raw material procurement costs. Third, a multi‐dimensional market cultivation strategy should be implemented. This strategy shapes the high‐end health image of the product by popularizing the health value of walnut meal (e.g., “rich in antioxidant polyphenols and high‐quality dietary fiber”). In addition, offline tasting and experience activities should be conducted to enhance consumer awareness with intuitive taste.

Although walnut meal demonstrates great potential in the vegetarian meat production industry owing to its excellent nutritional and functional properties, its practical application presents problems such as inferior flavor and texture, which require improvement. Thus, we propose the following targeted solutions: First, raw material compounding should be implemented to optimize the sensory quality. For the moisture extrusion process, soybean isolate protein can be replaced with pea protein. Furthermore, mushroom powder can be used for compound blending. Multiple protein sources and complementary flavored substances effectively enhance product flavor and improve sensory characteristics. Second, innovative technology should be implemented to improve product quality. This option involves the integration of high moisture extrusion, electrostatic spinning, and 3D printing techniques for targeted optimization of walnut meal texture and flavor. Third, interdisciplinary synergistic cooperation should be established. This option involves the integration of chemistry, biology, medicine, and engineering to develop a cross‐field research system, which promotes the utilization of walnut by‐products and product innovation applications. Finally, as sensory evaluation is crucial for the success of plant‐based products, future research could examine new sensory methods that combine quantitative and qualitative data. Techniques such as flavor profiling using trained panels and consumer acceptance analyses would offer a fuller picture of how products are perceived by the senses.

## 5 Summary

In summary, walnut protein sausages with unique walnut aroma, smooth surface, homogeneous texture, and firm structure were successfully developed for the first time in this study. Walnut meal was the main raw material, with soybean isolate protein and gluten as secondary components. Furthermore, high‐moisture extrusion technology was applied to the preparation of the protein sausages. However, walnut meal is not only a high‐quality source of plant protein, but it is also rich in various bioactive compounds, such as polyphenols, flavonoids, phytosterols, and antioxidants. Based on this, the paper suggests that these compounds should be the focus of future studies assessing the nutritional quality and stability of products. This research direction is highly significant. Firstly, it would make full use of the potential of walnut meal as a high‐quality plant protein source by clarifying the effects of its bioactive compounds on product nutrition. On the other hand, evaluating the impact of these compounds on product stability, such as their ability to inhibit oxidative deterioration and extend the shelf life of food products, could help to ensure the stability of functional foods during storage and processing. Thus, further research on the physicochemical properties and functional attributes of walnut meal is essential to comprehensively explore its application in the plant‐based food industry. This investigation provides a theoretical basis and practical guide for technological innovation in the plant‐based food industry.

## Author Contributions

**Ying Wang:** data curation (equal), methodology (equal), writing – original draft (equal). **Chengzhuang Lv:** data curation (equal), writing – original draft (equal). **Ping Zhang:** supervision (equal). **Fengzhong Wang:** supervision (equal). **Ting Zhang:** data curation (equal). **Yan Ma:** conceptualization (equal), funding acquisition (equal), writing – review and editing (equal). **Mingqiang Xu:** funding acquisition (equal), writing – review and editing (equal).

## Ethics Statement

This study does not involve any human or animal testing.

## Consent

Written informed consent was obtained from all study participants.

## Conflicts of Interest

The authors declare no conflicts of interest.

## Data Availability

The datasets used and/or analyzed in this study are available from the corresponding author upon reasonable request.

## References

[fsn370747-bib-0002] Bao, Y. , and P. Ertbjerg . 2019. “Effects of Protein Oxidation on the Texture and Water‐Holding of Meat: A Review.” Critical Reviews in Food Science and Nutrition 59, no. 22: 3564–3578. 10.1080/10408398.2018.1498444. 30040449

[fsn370747-bib-0003] Barak, S. , D. Mudgil , and B. S. Khatkar . 2014. “Biochemical and Functional Properties of Wheat Gliadins: A Review.” Critical Reviews in Food Science and Nutrition 55, no. 3: 357–368. 10.1080/10408398.2012.654863. 24915383

[fsn370747-bib-0004] Carballo, J. , N. Mota , G. Barreto , et al. 1995. “Binding Properties and Colour of Bologna Sausage Made With Varying Fat Levels, Protein Levels and Cooking Temperatures.” Meat Science 41, no. 3: 301–313. 10.1016/0309-1740(95)00001-2. 22060201

[fsn370747-bib-0005] Chen, F. L. , Y. M. Wei , B. Zhang , et al. 2010. “System Parameters and Product Properties Response of Soybean Protein Extruded at Wide Moisture Range.” Journal of Food Engineering 96, no. 2: 208–213. 10.1016/j.jfoodeng.2009.07.014.

[fsn370747-bib-0006] Chen, Y. , Y. Liang , F. Jia , et al. 2021. “Effect of Extrusion Temperature on the Protein Aggregation of Wheat Gluten With the Addition of Peanut Oil During Extrusion.” International Journal of Biological Macromolecules 166: 1377–1386. 10.1016/j.ijbiomac.2020.11.017. 33161084

[fsn370747-bib-0008] Golly, M. K. 2019. “Study on Preparation of Bioactive Peptides From Walnut ( *Juglans Regia* L.) Meal Using Ultrasonic Enhancement and the Coupling of Enzyme Hydrolysis and Membrane Separation.” JiangSu University. 10.27170/d.cnki.gjsuu.2019.000339.

[fsn370747-bib-0009] Handa, C. , S. Goomer , and A. Siddhu . 2011. “Physicochemical Properties and Sensory Evaluation of Fructoligosaccharide Enriched Cookies.” Journal of Food Science and Technology 49, no. 2: 192–199. 10.1007/s13197-011-0277-4. 23572841 PMC3550859

[fsn370747-bib-0010] Heinze, P. , and H. D. Isengard . 2001. “Determination of the Water Content in di Erent Sugar Syrups by Halogen Drying.” Food Control 12, no. 7: 483–486.

[fsn370747-bib-0011] Hou, Y. , and G. Wu . 2018. “Nutritionally Essential Amino Acids.” Advances in Nutrition 9, no. 6: 849–851. 10.1093/advances/nmy054. 30239556 PMC6247364

[fsn370747-bib-0012] Huang, G. L. , T. T. Liu , X. M. Mao , et al. 2023. “Insights Into the Volatile Flavor and Quality Profiles of Loquat (*Eriobotrya japonica* Lindl.) During Shelf‐Life via HS‐GC‐IMS, E‐Nose, and E‐Tongue.” Food Chemistry 20: 100886. 10.1016/j.fochx.2023.100886. PMC10739855 38144837

[fsn370747-bib-0014] Iizuka, K. 2021. “The Roles of Carbohydrate Response Element Binding Protein in the Relationship Between Carbohydrate Intake and Diseases.” International Journal of Molecular Sciences 22, no. 21: 12058. 10.3390/ijms222112058. 34769488 PMC8584459

[fsn370747-bib-0015] Kalač, P. 2013. “A Review of Chemical Composition and Nutritional Value of Wild‐Growing and Cultivated Mushrooms.” Journal of the Science of Food and Agriculture 93, no. 2: 209–218. 10.1002/jsfa.5960. 23172575

[fsn370747-bib-0016] Kazuo, H. 1995. “World Balance of Dietary Essential Amino Acids Relative to the 1989 Fao/who Protein Scoring Pattern.” Food and Nutrition Bulletin 16: 1–15. 10.1177/156482659501600210.

[fsn370747-bib-0017] Keerthana Priya, R. , A. Rawson , R. Vidhyalakshmi , et al. 2022. “Development of Vegan Sausage Using Banana Floret (*Musa Paradisiaca*) and Jackfruit (*Artocarpus heterophyllus* Lam.) as a Meat Substitute: Evaluation of Textural, Physico‐Chemical and Sensory Characteristics.” Journal of Food Processing and Preservation 46, no. 1: e16118. 10.1111/jfpp.16118.

[fsn370747-bib-0018] Kerr, W. L. , X. Wang , and S. G. Choi . 2005. “Physical and Sensory Characteristics of Low‐Fat Italian Sausage Prepared With Hydrated Oat.” Journal of Food Quality 28, no. 1: 62–77. 10.1111/j.1745-4557.2005.00010.x.

[fsn370747-bib-0019] Ketnawa, S. , and S. Rawdkuen . 2023. “Properties of Texturized Vegetable Proteins From Edible Mushrooms by Using Single‐Screw Extruder.” Food 12, no. 6: 1269. 10.3390/foods12061269. PMC10048080 36981195

[fsn370747-bib-0020] Kong Ling‐Ming, K. L. , L. F. Li Fang , Z. Y. Zong YuXia , et al. 2013. “Effect of Enzymatic Hydrolysis Conditions on Anti‐Oxidative Activity of Walnut Polypeptide.” Food Research and Development 34, no. 14: 106–109.

[fsn370747-bib-0022] Li, Y. , K. Yang , Z. He , et al. 2023. “Can Electronic Nose Replace Human Nose?─An Investigation of E‐Nose Sensor Responses to Volatile Compounds in Alcoholic Beverages.” ACS Omega 8, no. 18: 16356–16363. 10.1021/acsomega.3c01140. 37179643 PMC10173318

[fsn370747-bib-0023] Liu, Y. , Z. Qiao , Z. Zhao , et al. 2022. “Comprehensive Evaluation of Luzhou‐Flavor Liquor Quality Based on Fuzzy Mathematics and Principal Component Analysis.” Food Science & Nutrition 10, no. 6: 1780–1788. 10.1002/fsn3.2796. 35702309 PMC9179129

[fsn370747-bib-0024] Ma, X. , C. Zheng , Q. Zhou , et al. 2024. “Comparison Evaluation Pretreatments on the Quality Characteristics, Oxidative Stability, and Volatile Flavor of Walnut Oil.” Food Chemistry 448: 139124. 10.1016/j.foodchem.2024.139124. 38554586

[fsn370747-bib-0025] Mao, X. , Y. Hua , and G. Chen . 2014. “Amino Acid Composition, Molecular Weight Distribution and Gel Electrophoresis of Walnut ( *Juglans regia* L.) Proteins and Protein Fractionations.” International Journal of Molecular Sciences 15, no. 2: 2003–2014. 10.3390/ijms15022003. 24473146 PMC3958834

[fsn370747-bib-0026] Mozafarpour, R. , A. Koocheki , E. Milani , and M. Varidi . 2019. “Extruded Soy Protein as a Novel Emulsifier: Structure, Interfacial Activity and Emulsifying Property.” Food Hydrocolloids 93: 361–373. 10.1016/j.foodhyd.2019.02.036.

[fsn370747-bib-0027] Naqvi, S. N. , M. Liaquat , A. Kazmi , et al. 2024. “Sesame‐Enriched Delights: A Comparative Exploration of Physicochemical and Sensory Attributes in Fine and Whole Wheat Flour Cookies.” Food Science & Nutrition 12: fsn3.4343. 10.1002/fsn3.4343. PMC11521680 39479636

[fsn370747-bib-0028] Nishimura, K. , and H. Saeki . 2018. “Random‐Centroid Optimization Reveals the Strongest Superoxide Anion Radical Scavenging Activity of Maltose‐ and Ribose‐Conjugated Chicken Myofibrillar Protein.” Food Science and Technology Research 24, no. 3: 551–557. 10.3136/fstr.24.551.

[fsn370747-bib-0029] Noguerol, A. T. , V. Larrea , and M. J. Pagán . 2022. “The Effect of Psyllium ( *Plantago ovata* Forsk) Fibres on the Mechanical and Physicochemical Characteristics of Plant‐Based Sausages.” European Food Research and Technology 248, no. 10: 2483–2496. 10.1007/s00217-022-04063-2. 35818621 PMC9261230

[fsn370747-bib-0030] Osen, R. , S. Toelstede , F. Wild , et al. 2014. “High Moisture Extrusion Cooking of Pea Protein Isolates: Raw Material Characteristics, Extruder Responses, and Texture Properties.” Journal of Food Engineering 127: 67–74. 10.1016/j.jfoodeng.2013.11.023.

[fsn370747-bib-0031] Pietrasik, Z. , and O. P. Soladoye . 2021. “Functionality and Consumer Acceptability of Low‐Fat Breakfast Sausages Processed With Non‐Meat Ingredients of Pulse Derivatives.” Journal of the Science of Food and Agriculture 101, no. 11: 4464–4472. 10.1002/jsfa.11084. 33432585

[fsn370747-bib-0032] Sato, K. , S. Ueno , and J. Yano . 1999. “Molecular Interactions and Kinetic Properties of Fats.” Progress in Lipid Research 38, no. 1: 91–116. 10.1016/S0163-7827(98)00019-8. 10396603

[fsn370747-bib-0033] Sha, L. , and Y. L. Xiong . 2020. “Plant Protein‐Based Alternatives of Reconstructed Meat: Science, Technology, and Challenges.” Trends in Food Science & Technology 102: 51–61. 10.1016/j.tifs.2020.05.022.

[fsn370747-bib-0034] Sowbhagya, H. B. , P. F. Suma , S. Mahadevamma , and R. N. Tharanathan . 2007. “Spent Residue From Cumin–A Potential Source of Dietary Fiber.” Food Chemistry 104, no. 3: 1220–1225. 10.1016/j.foodchem.2007.01.066.

[fsn370747-bib-0035] Szpicer, A. , A. Onopiuk , M. Barczak , et al. 2022. “The Optimization of a Gluten‐Free and Soy‐Free Plant‐Based Meat Analogue Recipe Enriched With Anthocyanins Microcapsules.” LWT 168: 113849. 10.1016/j.lwt.2022.113849.

[fsn370747-bib-0036] Tang, H. , C. Xu , Y. Jiang , et al. 2021. “Evaluation of Physical Characteristics of Typical Maize Seeds in a Cold Area of North China Based on Principal Component Analysis.” PRO 9, no. 7: 1167. 10.3390/pr9071167.

[fsn370747-bib-0037] Vinayashree, S. , and P. Vasu . 2021. “Biochemical, Nutritional and Functional Properties of Protein Isolate and Fractions From Pumpkin ( *Cucurbita moschata* var. Kashi Harit) Seeds.” Food Chemistry 340: 128177. 10.1016/j.foodchem.2020.128177. 33002826

[fsn370747-bib-0038] Wójtowicz, A. , K. Lisiecka , M. Mitrus , et al. 2019. “Physical Properties and Texture of Gluten‐Free Snacks Supplemented With Selected Fruit Additions.” International Agrophysics 4, no. 33: 407–416. 10.31545/intagr/112563.

[fsn370747-bib-0039] Wu, P. , J. Zhou , Q. Deng , et al. 2024. “Structure, Nutritional Value, Extraction, Functional Properties of Walnut Proteins and Their Application in Foods: A Review.” Food Science 45, no. 15: 329–337.

[fsn370747-bib-0040] Xie, Y. , N. Jiang , H. Su , et al. 2023. “Effect of Functional Lipids on the Quality of Walnut Butter Prepared From Defatted Walnut Meal by Ball Mill Grinding.” 10.22541/au.167353928.80753390/v1.

[fsn370747-bib-0041] Xin, L. , L. Guo , S. Edirs , et al. 2022. “An Efficient Deacidification Process for Safflower Seed Oil With High Nutritional Property Through Optimized Ultrasonic‐Assisted Technology.” Molecules 27, no. 7: 2305. 10.3390/molecules27072305. 35408704 PMC9000557

[fsn370747-bib-0042] Xu, J. , X. Xu , Z. Yuan , et al. 2022. “Effect of Hemp Protein on the Physicochemical Properties and Flavor Components of Plant‐Based Yogurt.” LWT 172: 114145. 10.1016/j.lwt.2022.114145.

[fsn370747-bib-0043] Xu, X. , X. Zhang , M. Sun , et al. 2023. “Optimization of Mixed Fermentation Conditions of Dietary Fiber From Soybean Residue and the Effect on Structure, Properties and Potential Biological Activity of Dietary Fiber From Soybean Residue.” Molecules 28, no. 3: 1322. 10.3390/molecules28031322. 36770993 PMC9920189

[fsn370747-bib-0044] Yang, Y. , D. Pan , Y. Wang , et al. 2020. “Effect of Reconstituted Broth on the Taste‐Active Metabolites and Sensory Quality of Stewed and Roasted Pork‐Hock.” Food 9, no. 2: 13. 10.3390/foods9040513. PMC7230635 32326064

[fsn370747-bib-0045] Zhang, M. , H. Liu , and Q. Wang . 2022. “Characterization of β‐Glucan‐Peanut Protein Isolate/Soy Protein Isolate Conjugates and Their Application on Low‐Fat Sausage.” Molecules 27, no. 9: 3037. 10.3390/molecules27093037. 35566387 PMC9099641

[fsn370747-bib-0046] Zhu, Y. , Y. Zhang , and Z. Peng . 2021. “Effect of Eggplant Powder on the Physicochemical and Sensory Characteristics of Reduced‐Fat Pork Sausages.” Food 10, no. 4: 743. 10.3390/foods10040743. PMC8067279 33915964

